# Water Activity Effect on Microbial Behavior During Hyperbaric Storage at Room Temperature of Watermelon Juice as a Case Study

**DOI:** 10.3390/foods15040741

**Published:** 2026-02-17

**Authors:** Vasco Lima, Carlos A. Pinto, Jorge A. Saraiva

**Affiliations:** 1 Associated Laboratory for Green Chemistry—Clean Technologies and Processes (LAQV-REQUIMTE), Department of Chemistry, University of Aveiro, 3810-193 Aveiro, Portugal; vasco.lima@ua.pt (V.L.); carlospinto@ua.pt (C.A.P.); 2 CBQF—Centro de Biotecnologia e Química Fina—Laboratório Associado, Escola Superior de Biotecnologia, Universidade Católica Portuguesa, Rua Diogo Botelho 1327, 4169-005 Porto, Portugal

**Keywords:** watermelon juice, hyperbaric storage, water activity, microbial growth control, microbial inactivation, Weibull modelling

## Abstract

Hyperbaric storage (HS) is a novel technology for storing foods under mild pressures that, when used at room temperature (RT), offers much lower energy costs and greenhouse gas emissions than conventional refrigeration (RF). Watermelon juice (WJ), with interesting associated health benefits, is highly perishable due to its pH (5.20–6.70) and water activity (a_W_, 0.97–0.99). This work investigated a_W_’s impact on WJ’s preservation by HS/RT, studying the behavior of *Escherichia coli*, *Listeria monocytogenes*, and *Saccharomyces cerevisiae* inoculated in WJ at a_W_ 0.930–0.971 stored at 25–75 MPa for up to 28 days, along with RT and RF atmospheric pressure controls. The results showed that HS could control microbial growth, and, during storage, inactivation was also observed, and that HS’s impact depended on the a_W_ level, microorganism, and storage pressure. Inactivation was often increased at 50–75 MPa and at a_W_ 0.930–0.950, while growth mostly occurred at a_W_ 0.971. The inactivation curves were mathematically described by the first-order and Weibull kinetic models, with the Weibull model frequently obtaining better fits. These findings support HS’s potential for food preservation, showing better overall WJ growth control and inactivation effects than RF, without temperature control, making HS environmentally friendlier.

## 1. Introduction

Hyperbaric storage (HS) is a novel food preservation methodology consisting of storing foods under mild pressure levels (up to 100–125 MPa) for lengthy periods (days, weeks, or months at room-like temperature (RT). HS has been proposed as an alternative to conventional refrigeration (RF), and recent studies have shown that it can increase microbial safety and increase the shelf-life of perishable products. When used at RT, HS allows lower energy consumption since energy is used only for the pressurization stage, and the vessel sealing can maintain the pressure level during storage without needing additional energy [1].

Several studies have shown that during the HS of perishable foods such as meat, fish, milk, and related products, ready-to-eat foods, and fruit juices, microbial growth control and inactivation could be obtained (often to levels over 6 log units). The microbial behavior is impacted by the storage conditions (pressure level, temperature, and duration), the type of microorganism, the food composition, and parameters like the water activity (a_W_) and pH, amongst others [2]. A high a_W_ has often been associated with increased microbial inactivation in most nonthermal processing technologies, including high-pressure processing (HPP). The increased pressure resistance at lower a_W_ values is attributed to the increased stability of macromolecules due to the decreased cell compressibility produced by the higher concentration of solutes in the cytoplasm of bacterial cells [3].

Known for its attractive color, flavor, and fresh aroma, watermelon juice (WJ) is popular amongst consumers worldwide [4]. It is associated with health benefits related to its interesting nutritional composition that includes a high water content, vitamins (A, B, C, and E), minerals (K, Mg, Ca, and Fe), sugars, specific amino acids (citrulline and arginine), and bioactive compounds (like carotenoids and phenolic compounds) [5,6]. However, WJ’s a_W_ (0.97–0.99) and (pH 5.2–6.7), make this juice highly perishable due to facilitated microbial development and enzymatic deterioration [7].

To the best of the authors’ knowledge, systematic research concerning the impact of a_W_ during HS is not reported in the literature, so this work aimed at evaluating how a_W_ variation affects the behavior of microorganisms inoculated in raw WJ preserved by HS (25–75 MPa) at RT (18–23 °C). Three microorganisms were used in this study, *Escherichia coli* (a surrogate for the pathogenic *E. coli* O157:H7) and *Listeria monocytogenes* were chosen for their relevance to food safety and to represent Gram-negative and Gram-positive bacteria, since differences in pressure resistance have been demonstrated between these two groups [8], while *Saccharomyces cerevisiae* was selected due to the relevance of the issues caused by its spoilage activity in foods and the likelihood to be found in WJ [7,9].

## 2. Materials and Methods

### 2.1. Watermelon Juice Preparation

Mature seeded red watermelons (*Citrullus lanatus*) acquired from a local supermarket were stored at 4 °C until washing, peeling, crushing, and filtration with a sterilized cotton filter in sterile conditions in a laminar flow cabinet (BioSafety Cabinet Telstar Bio II Advance, Terrassa, Spain) to prevent contaminations and the resulting WJ was either used immediately or frozen in 100 mL aliquots and kept at −20 °C until use.

Prior to each experiment, the WJ was thawed at 4 °C overnight and equilibrated at 25 °C for 3 h. WJ’s pH was adjusted from 5.60–5.78 (fresh juice pH values) to 6.50 with a NaOH solution using a properly calibrated glass electrode (pH electrode 50 14, Crison Instruments, S.A., Barcelona, Spain) at 25 °C. This occurred in the context of a broader set of results under publication from experiments related to the impact of pH variation in microbial behavior in WJ preserved by HS/RT, as it was observed that pH 6.50 was the pH level that would benefit most from HS preservation due to being the most perishable level studied overall.

WJ’s a_W_ values were measured with an a_W_ meter (Rotronic HygroLab., Ettlingen, Germany) at 20 °C. The juice’s initial a_W_ was 0.971 ± 0.001, and to lower the a_W_ to 0.950 and 0.930, sucrose (LabChem, Loures, Portugal) was added, this being the quantity of sucrose necessary calculated through the linear regression of the measured a_W_ values (Table S1 and Figure S1). Sucrose was used since it is a solute already present in the juice’s composition. The WJ was then sterilized at 121 °C for 15 min, inoculated with the microorganism, and divided into 0.4 mL micro test tubes with cap type Beckman^®^ (Kartell Labware, Noviglio, Italy).

In addition to a_W_ 0.971, the value measured for the WJ used in this study, representative of a typical WJ a_W_ level and the most perishable, the other two a_W_ levels were chosen since, despite still being high a_W_ levels for food preservation, they represent increasing hindrance in the ability to control microbial growth, reducing the juice’s perishability. These levels also allow us to compare the behavior of the microorganisms studied, since the bacteria used are expected to show hindrances in growth as the a_W_ is lowered and the WJ becomes less perishable, especially below a_W_ 0.950, the general lower limit for growth shown by many bacteria, while yeasts generally have a lower a_W_ to hinder their development.

### 2.2. Hyperbaric Storage Experiments

HS equipment (FPG13900, Stansted Fluid Power, Stansted, UK) equipped with three pressure vessels each with an inner diameter of 30 mm and a height of 500 mm (400 mL capacity each) was used to perform the HS experiments, at RT (18–23 °C), using a propylene glycol and water mixture (40:60, *v*/*v*) was used as pressurization fluid.

The micro test tubes containing the inoculated juice were placed in small, low-permeability polyamide/polyethylene bags with a thickness of 90 µm (Ideiapack—Comércio de embalagens Lda, Abraveses, Viseu, Portugal) and stored for up to 28 days at different pressure levels (25–75 MPa). Control samples at atmospheric pressure (AP, 0.1 MPa) were kept in the dark at RT (AP/RT) or under refrigerated conditions (AP/RF, ~4 °C).

### 2.3. Microbiological Analyses

#### 2.3.1. Preparation of the Inoculated Microorganisms

A non-pathogenic surrogate strain of *E. coli* ATCC 25922, the pathogenic *Listeria monocytogenes* FSL J1-177 1/2b, and *Saccharomyces cerevisiae* were used for this work. Pure cultures of *E. coli* and *L. monocytogenes* were previously grown in Tryptic Soy Broth (Liofilchem, Roseto degli Abruzzi, Italy) at 37 °C overnight and for 24 h, respectively, in an incubating orbital shaker (VWR, Carnaxide, Portugal) at 150 rpm. *S. cerevisiae* was isolated from baker’s yeast (Condi, Camarate, Portugal) acquired from a local supermarket: baker’s yeast was plated in Yeast Malt Agar medium (YMA, HiMedia, Maharashtra, India) and incubated for 3 days at 30 °C, then, a single colony was inoculated in 200 mL of Yeast Malt Broth (HiMedia, Mumbai, India), followed by incubation at 30 °C for 3 days under orbital agitation (150 rpm) in the orbital shaker. Baker’s yeast was used since it is easily obtained and could be used as a model. Glycerol (10% *v*/*v*) (Biochem Chemopharma, Cosne-Cours-sur-Loire, France) was added to the grown microorganisms’ stocks to act as a cryoprotectant agent, and the stocks were divided into 2 mL aliquots in microtubes (Deltalab, S. L., Barcelona, Spain) and frozen at −80 °C until needed. After thawing at RT, the necessary micro tubes were centrifuged for 10 min at 6000× *g*, the supernatant was discarded, while the remaining mass of cells (pellet) was used for the inoculation.

#### 2.3.2. Quantification of Microorganisms

After each experiment, WJ samples were serially diluted in Ringer’s solution and plated on the appropriate media in accordance with the Miles and Misra method [10]. *E. coli* and *L. monocytogenes* were enumerated in Tryptic Soy Agar medium (VWR International, Radnor, PA, USA) after incubating for 24 h at 37 °C, while *S. cerevisiae* was enumerated in YMA after incubating for 36 h at 30 °C. Petri dishes containing 10–100 CFU were chosen for counting and results were expressed as log CFU/mL of WJ and presented as microbial log load variation (log (*N*/*N_0_*)), calculated by the log load difference between the microbial load at each storage sampling point (N), and the initial microbial load at the start of the experiments (*N_0_*). The detection limit (DL) was 1.70 log CFU/mL when no counts were found in the lowest dilution (i.e., when the WJ was directly plated), as stated in the Miles and Misra method [10], while the quantification limit (QL) was 2.70 log CFU/mL when only 1–10 colonies were counted in the lowest serial dilution [11].

#### 2.3.3. Microbial Inactivation Kinetic Modelling

The microbial inactivation kinetics parameters were estimated with two mathematical models (first-order and Weibull) when enough data points existed to apply these models to the inactivation of microorganisms. The first-order model is often employed in the food industry, while the Weibull model was selected as it is one of the most used nonlinear models and has good fits for microbial inactivation by HPP [12]. The data was fitted with Matlab R2023a (MathWorks Inc., Natick, MA, USA) using the Trust-region nonlinear regression method, considering only quantifiable experimental values (i.e., values greater than the QL). The root mean square error (*RMSE*), the coefficient of determination (*R*^2^), and the adjusted *R*^2^ (*Adj-R*^2^) were estimated to assess the quality of the fit for each model, while an *RMSE* value close to 0 and an *Adj-R*^2^ close to 1 indicate the adequacy of the model to describe the results. The kinetic parameter values obtained were presented as value ± 95% confidence interval.

Equation (1) presents the first-order linear model that considers that, in the case of microbial inactivation by pressure, the *N_0_* of microbial population is diminished to *N* after some storage time, *t*, at a constant pressure. Equation (1) was used to calculate the decimal reduction time (*D_p_*, in days), which is the time necessary to achieve a decimal reduction in microbial load [13], and only *D_p_*-values estimated with an *Adj-R*^2^ > 0.900 were presented.

(1) $$\mathrm{Log}(\frac{N}{N0})=-\frac{t}{Dp}$$

In Equation (2), where the Weibull model is presented, *b* represents the microbial inactivation rate constant, and *n* stands for the shaping factor. An upward concave curve is shown when *n* < 1, displaying the adaptation of a microbial population that has moderate or high resistance to the applied pressure, with gradual count reductions as time passes. However, a downward concave curve appears in the case of *n* > 1, signaling quicker inactivation brought about by the cumulated damage produced by pressure, while *n* = 1 results in first-order kinetics [12].

(2) $$Log(\frac{N}{N0})=-b*t^{n}$$

### 2.4. Statistical Analysis

The experiments were performed with three different replicates (samples), each plated in duplicate. One-way analysis of variance (ANOVA) followed by the post hoc Tukey’s test was conducted to assess the differences for each curve at different storage times, with the level of significance set at 5%. Kinetic parameters were used to identify differences between the same pressure level at different a_W_ levels or between distinct pressures at the same a_W_ level, based on the 95% confidence interval of each one.

## 3. Results and Discussion

### 3.1. Microbial Analyses

#### 3.1.1. *Escherichia coli*

**Figure 1:**
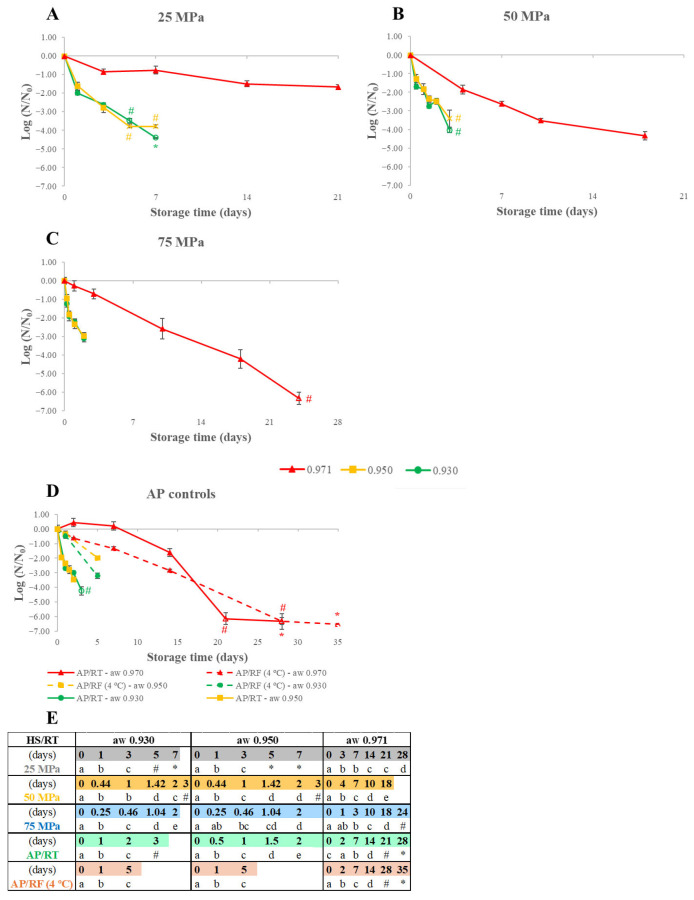
*Escherichia coli* load variation for watermelon juice at a_W_ 0.930, 0.950, and 0.971 preserved by hyperbaric storage (HS) at uncontrolled room temperature (RT, 18–23 °C) at different pressure levels of 25 MPa (**A**), 50 MPa (**B**), 75 MPa (**C**) and atmospheric pressure controls (**D**) at RT (AP/RT) or refrigerated (AP/RF, 4 °C) conditions. Results are expressed in log CFU/mL, and initial loads are available in Table 1. Different letters in Table (**E**) denote significant differences (*p* < 0.05) between storage days for each storage condition at a given a_W_ level (a–e). Unfilled symbols represent counts that are below the quantification (marked by #) or detection (marked by *) limits of 2.7 and 1.7 log CFU/mL, respectively.

The impact of varying the a_W_ of WJ preserved by HS on *E. coli*’s behavior was evaluated at a_W_ 0.930, 0.950, and 0.971 and pressures ranging from 25 MPa to 75 MPa is shown in Figure 1, as well as the statistical differences within each curve. Table 1 details the initial *E. coli* loads (*N_0_*), and the differences in the initial *E. coli* loads for the a_W_ 0.971 results relate to having used this data from a larger set of unpublished PhD results and from the decision made to employ higher initial loads after having observed the first results with lower initial loads.

Figure 1 shows that *E. coli*’s growth control and inactivation were achieved during HS at all pressure and a_W_ levels evaluated, unlike what was observed with the AP/RT control at a_W_ 0.971, which exhibited growth after one week of storage. The impact of lowering the a_W_ was clear, as inactivation rates for a_W_ 0.971 were the lowest at any pressure level examined (Figure 1). At 75 MPa, for example, it took 2 days to inactivate around 3 log CFU/mL at the a_W_ 0.930 and 0.950 levels, whereas 10 days were required to reduce the counts in 2.58 log CFU/mL at a_W_ 0.971. Additionally, the a_W_ 0.930 and 0.950 curves were very similar for the three pressure levels studied (Figure 1A–C) and a similar result was verified for the AP/RT control for a_W_ 0.930 and 0.950 curves (Figure 1D), while inactivation was faster for AP/RF samples at a_W_ 0.930 compared to 0.950, with 3.20 and 1.98 logs reductions, respectively, after 5 days.

The a_W_ 0.930–0.950 levels, AP/RT, showed the lowest inactivation rates, followed by the samples stored at 25 MPa, and, with minor differences, the other curves, in which the AP/RF and 75 MPa samples appeared to allow slightly higher inactivation rates. For the a_W_ 0.971 level, results suggest differences in the AP/RT and 25 MPa samples, as a minor growth was noted for the AP/RT WJ (followed by a sharp reduction after) and storage at 25 MPa achieved the smallest reduction effects (1.97 log CFU/mL after 28 days) with increased separation from the other curves, while WJ stored at 50 and 75 MPa showed the highest inactivation rates.

*E. coli* inactivation values measured at the end of the HS ranged from 1.97 log CFU/mL (a_W_ 0.971 WJ after 28 days at 25 MPa) and, at least, 6.31 log CFU/mL (a_W_ 0.971 WJ after 24 days at 75 MPa). In some cases (especially for a_W_ 0.971), reductions were near or above the level of pasteurization of 5 log CFU/mL defined by the U.S. Food and Drug Administration (FDA) for the most resistant microorganism that poses a threat to public health [14,15], although for a_W_ 0.930–0.950 the shape of the curves indicate that a 5 log CFU/mL reduction could be achieved if storage time was prolonged. For these two a_W_ levels, it is worth noting that even a pressure as low as 25 MPa caused about 4 log CFU/mL after 7 days, and, at 75 MPa, reductions over 3 log units were observed in 2 days.

The increased inactivation at the lowest a_W_ levels observed in Figure 1 is consistent with expectations, as *E. coli*’s minimum a_W_ limit for growth is 0.950 [16], meaning that, even at a_W_ 0.950, its ability to grow may be somewhat hindered and its inactivation may be promoted and this is corroborated by the only instance of growth having occurred at a_W_ 0.971 (AP/RT). Although increased inactivation by pressure treatments is expected at higher a_W_ levels, due to the lowered pressure resistance [17], the results suggest that lowering the a_W_ had a stronger effect and that, perhaps, the differences in a_W_ between 0.930 and 0.950 were not sufficient to observe the pressure effect.

Increased inactivation rates were expected as pressure levels increased, and this was partially seen as inactivation at 25 MPa was slower than at 50 and 75 MPa. However, at those two latter pressures, differences were less clear, since curves overlapped in some instances. *E. coli* inactivation in foods preserved by HS has been reported for WJ (pH 6.23), when the juice was stored up to 10 days at 50–100 MPa/RT and after 3 days at 75–100 MPa, *E. coli* counts reached levels below the DL, with reductions of, at least, 2.10 log CFU/mL, while at 50 MPa, a decrease of 1 log CFU/mL was noticed after the first 3 days and, at the 6th day counts reached levels below the DL [18].

The effect of increased inactivation rates at higher pressure levels has also been demonstrated in raw milk (pH 6.68) preserved by HS/RT (50–100 MPa for up to 31 days) that showed *E. coli* reductions of, at least, 4.07 log CFU/mL after 21 days at 50 MPa or 10 days at 75 MPa [19]; and milk (pH 6.73) pasteurized by HPP (two consecutive cycles at 600 MPa for 90 and 120 s, respectively) preserved by HS/RT (50–100 MPa for up to 120 days) that, after 21 days, revealed the inactivation of, at least, 6.68 log CFU/mL at 75 MPa and 2–3 log CFU/mL at 50 MPa [20].

Although there is no published HS study relating to a_W_ variation, some authors investigated its influence on *E. coli* inactivation by HPP, reporting mixed results. The first study treated sugar-cookie dough at different a_W_ (0.80–0.87) at 100–600 MPa for 1–6 min at 22 °C and observed that the highest inactivation was measured at a_W_ 0.83 with a 1.4 log CFU/g reduction [21], supporting the results in Figure 1. However, a work performed with *E. coli* k12 inoculated in solid model gels (made of distilled water, glycerol, kelcogel (Gellan gum), and CaCl_2_) or in liquid models (glycerol and solute like sorbitol, fructose, or NaCl) at a_W_ 0.90–0.99 applied a HPP treatment (400–600 MPa, 1–10 min, 4–20 °C) and observed that the maximum inactivation occurred at a_W_ 0.99 with reductions of 6–7 log CFU/mL (depending on the temperature) [22]. The enhanced pressure resistance obtained by lowering the a_W_ is attributable to the increased stability gained by macromolecules because of the decreased cell compressibility caused by the increased concentration of solutes in the cytoplasm of bacterial cells [3].

Figure 1 shows that, during HS, *E. coli* growth was not detected at any a_W_ or storage pressure level tested, that inactivation also occurred, and that these results can be attributed to both HS and lowering the a_W_. However, considerable load reductions were also observed in the AP controls, especially at RT (at all a_W_ levels) and for RF at a_W_ 0.971, often higher than the reduction obtained for HS at the lower pressure levels, indicating a protective effect of pressure at the lower pressure levels studied.

#### 3.1.2. *Listeria monocytogenes*

**Figure 2:**
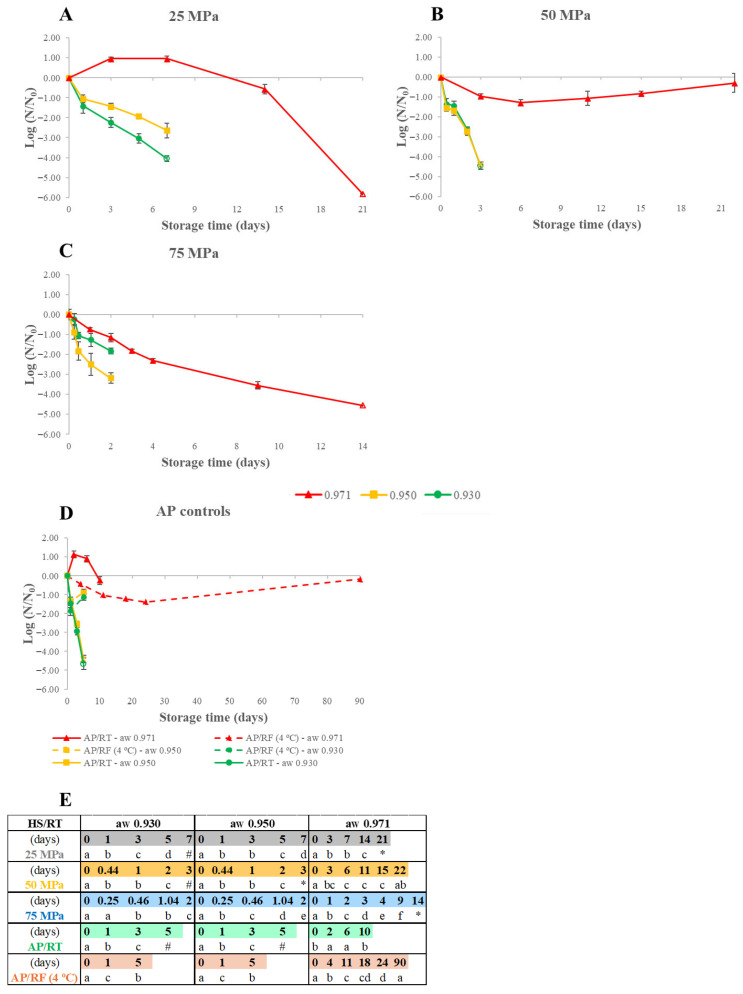
*Listeria monocytogenes* load variation for watermelon juice at a_W_ 0.930, 0.950, and 0.971 preserved by hyperbaric storage (HS) at uncontrolled room temperature (RT, 18–23 °C) at different pressure levels of 25 MPa (**A**), 50 MPa (**B**), 75 MPa (**C**) and atmospheric pressure controls (**D**) at RT (AP/RT) or refrigerated (AP/RF, 4 °C) conditions. Results are expressed in log CFU/mL, and initial loads are available in Table 2. Different letters in Table (**E**) denote significant differences (*p* < 0.05) between storage days for each storage condition at a given a_W_ level (a–f). Unfilled symbols represent counts that are below the quantification (marked by #) or detection (marked by *) limits of 2.7 and 1.7 log CFU/mL, respectively.

Figure 2 shows the results of HS’s impact on *L. monocytogenes* inoculated in WJ at a_W_ 0.930, 0.950, or 0.971 stored at 25–75 MPa/RT conditions, as well as the AP controls and the statistical analysis for variations within each curve. Initial *L. monocytogenes* loads are detailed in Table 2.

HS controlled the growth of *L. monocytogenes* at all pressures tested for a_W_ 0.930–0.950, and inactivation was also observed, even to levels below the QL or DL. However, for a_W_ 0.971 WJ, growth throughout storage was only controlled for the WJ stored at 75 MPa, with inactivation occurring from the beginning, and the lowest inactivation rates were observed. For example, after 2 days of storage at 75 MPa, reduction values of 1.83, 3.18, and 1.15 log CFU/mL were obtained for WJ at a_W_ 0.930, 0.950, and 0.971, respectively.

This data for WJ at a_W_ 0.930 and 0.950 was similar for every pressure level and AP control tested, with all cases showing inactivation during storage. The most-evident differences are the increased inactivation rate obtained at 75 MPa and the fact that, at a_W_ 0.930, curves overlap until the 2nd storage day, unlike at a_W_ 0.950, in which distinct inactivation rates are seen from the start. However, even at a_W_ 0.971, the most perishable level tested and even at the lowest pressure, 25 MPa, HS was more effective in inactivating *L. monocytogenes* than the AP/RF control, and this is especially visible and faster at 75 MPa. Unexpectedly, at 50 MPa, the curve does not follow the same trend as at 25 and 75 MPa, showing an inactivating trend up until day 6 with a 1.30 log CFU/mL reduction, while after that, growth occurred until day 22, showing a 0.30 log units’ reduction compared to the initial inoculated load, with the reason for this being unknown.

Figure 2 displays cases of increased inactivation at the lowest a_W_ levels, which are not consistent with initial expectations since the minimum a_W_ limit for the growth of *L. monocytogenes* is 0.92 [16], indicating that all a_W_ levels tested should allow its growth (especially for AP controls). Furthermore, higher pressure resistance is generally expected at lower a_W_ levels, limiting the inactivation effects of HPP treatments [17]. These expectations were not met since growth was only seen at a_W_ 0.971, and inactivation was enhanced at a_W_ 0.930 and 0.950, implying that lowering the a_W_ had a greater impact on *L. monocytogenes*’ behavior than the expected heightened pressure resistance. Overall, the inactivation values recorded in the HS period varied between 1.83 log CFU/mL (a_W_ 0.930 WJ stored at 75 MPa for 2 days) and at least 5.85 log CFU/mL (a_W_ 0.971 WJ after 21 days at 25 MPa).

There are no available HS works on the variation of a_W_, but some authors have tested different a_W_ levels during HPP treatments, focusing on *L. monocytogenes* inactivation. Their findings showed that the maximum inactivation recorded was at the highest a_W_ level examined, or, if not at the highest level, it occurred close to this. One example is the work performed in different solutions of sodium chloride (0.2–5.0 M), sucrose (0.9–2.0 M), and sodium phosphate buffer (0.01–1.0 M) at a_W_ levels between 0.75–1.0 treated by HPP (400 MPa, 10 min, 25 °C), with the authors reporting that the maximum inactivation occurred at a_W_ 1.0 (~8 log CFU/mL) [23]. Another work tested the HPP treatment (600 MPa, 5 min, 18–20 °C) of peptone water/glycerol solutions (a_W_ 0.10–0.99), discovering maximum inactivation at a_W_ 0.86–0.99 with 5.5–6.5 log CFU/mL inactivated [24]. Similar findings have been reported for dry-cured hams [25], Spanish chorizo sausage [26], cheese and cheese-distilled water mixtures [27], and two meat models [3,28].

There is no published HS research on *L. monocytogenes,* but *L. innocua* has often been used as its surrogate. Increased *L. innocua* inactivation rates caused by rising storage pressure levels have been reported in different foods. For WJ (pH 6.73) stored at 50–100 MPa up to 10 days at RT (18–23 °C), *L. innocua* counts were reduced to levels below the DL, inactivating, at least, 2.55 log CFU/mL after 6 days at 75 MPa or 10 days at 100 MPa; however, at 50 MPa, a growth of over 1 log CFU/mL was observed after 10 days [18]. Similarly, in the present work, for a_W_ 0.971, only at 75 MPa did growth and inactivation occur. Other foods in which this effect has been described include raw milk [19], HPP-pasteurized (two consecutive cycles at 600 MPa for 90 and 120 s, respectively) milk [29], salmon [30], raw minced bovine meat, and raw pork in pieces [31].

Figure 2’s results reveal that, besides no growth occurring during HS/RT preservation of WJ at a_W_ 0.930 and 0.950 WJ, for WJ at a_W_ 0.971 WJ kept at 75 MPa, inactivation was even observed during storage. At a_W_ 0.971, reductions were sometimes near or above the FDA’s pasteurization level of 5 log CFU/mL [14,15]. These findings can be linked to the impact of lowering the a_W_ and the effect of HS on *L. monocytogenes*’ behavior. However, for a_W_ 0.971 WJ stored at AP/RT or 25 MPa/RT conditions where a small growth occurred before a sharp inactivating trend, additional inactivation may have occurred due to increased nutrient consumption caused by the higher initial load used in the present work, which may have resulted in nutrient scarcity and bacterial death.

#### 3.1.3. *Saccharomyces cerevisiae*

**Figure 3:**
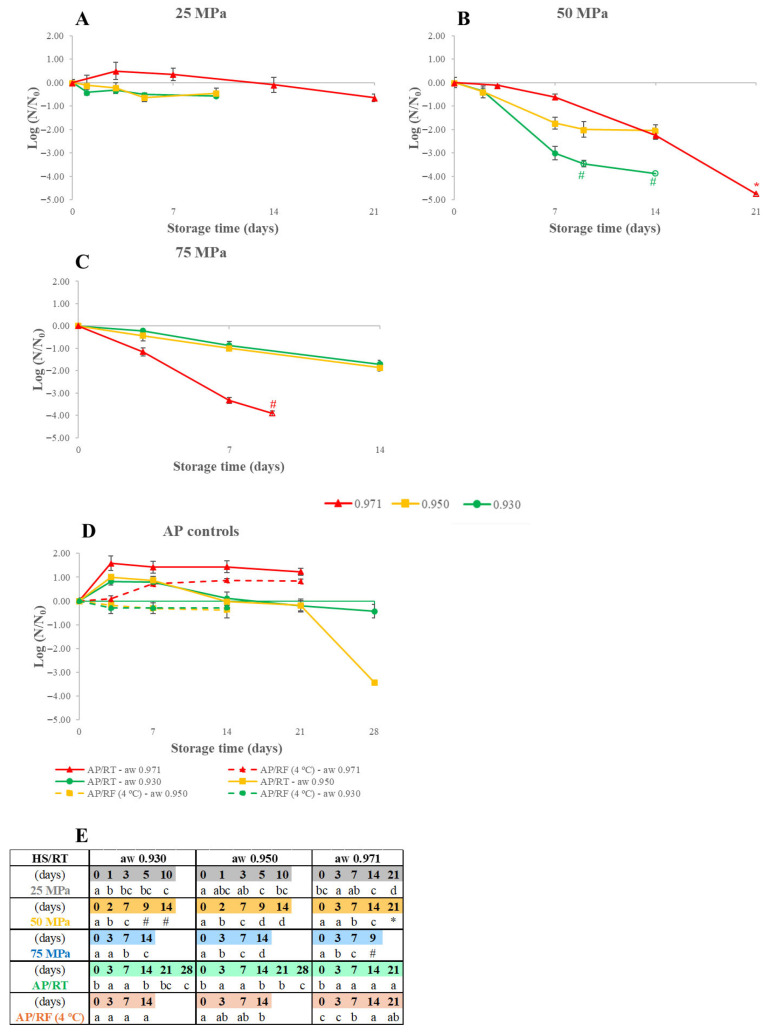
*Saccharomyces cerevisiae* load variation for watermelon juice at a_W_ 0.930, 0.950, and 0.971 preserved by hyperbaric storage (HS) at uncontrolled room temperature (RT, 18–23 °C) at different pressure levels of 25 MPa (**A**), 50 MPa (**B**), 75 MPa (**C**), and atmospheric pressure controls (**D**) at RT (AP/RT) or refrigerated (AP/RF, 4 °C) conditions. Results are expressed in log CFU/mL, and initial loads are available in Table 3. Different letters in Table (**E**) denote significant differences (*p* < 0.05) between storage days for each storage condition at a given a_W_ level (a–d). Unfilled symbols represent counts that are below the quantification (marked by #) or detection (marked by *) limits of 2.7 and 1.7 log CFU/mL, respectively.

The behavior of *S. cerevisiae* inoculated in WJ preserved by HS/RT was also investigated, and the results regarding the effect of varying WJ’s a_W_ (0.930, 0.950, and 0.971) and the storage pressure (25–75 MPa) are shown in Figure 3, along with the AP controls and the statistical analysis for differences within each curve. Table 3 presents the initial *S. cerevisiae* load inoculated in the WJ.

Figure 3 reveals that HS could control the growth of *S. cerevisiae* and cause its inactivation, even to levels below the QL or DL, at every a_W_ and storage pressure level tested, apart from a_W_ 0.971 WJ stored at 25 MPa, which showed minor growth until the 14th day. The AP/RF samples at a_W_ 0.930 and 0.950 were shown to successfully control the growth (Figure 3D), while samples at AP/RT showed growth, at peak values, of 0.82, 1.00, and 1.58 log CFU/mL for a_W_ 0.930, 0.950, and 0.971, respectively.

Figure 3 shows that the biggest difference between the curves of the three a_W_ levels occurred at 75 MPa, as a_W_ 0.971 allowed higher inactivation rates only at this pressure level. At 75 MPa, a_W_ 0.971 WJ showed a decrease in counts of at least 3.91 log CFU/mL after 9 days, while, after 14 days, only 1.70 and 1.87 log CFU/mL for a_W_ 0.930 and 0.950, respectively, were inactivated. Interestingly, with an increase in pressure, differences between the a_W_ levels were revealed: at 25 MPa, a_W_ 0.971 allowed growth before a small load reduction was seen, and the a_W_ 0.930 and 0.950 curves were similar; at 50 MPa, whereas a_W_ 0.971 remained the level with the slowest reductions, differences between a_W_ 0.930 and 0.950 were seen (2.24 and, at least, 3.88 log CFU/mL inactivated after 14 days for levels 0.930 and 0.950, respectively); and, at 75 MPa, while the a_W_ 0.930 and 0.930 curves were similar again, a_W_ 0.971 now allowed the fastest reductions.

Distinct *S. cerevisiae*’s behavior patterns were seen in WJ with a_W_ 0.971 WJ and a_W_ 0.930–0.950 in Figure 3. At a_W_ 0.971, the behavior matches expectations, since increased inactivation rates were observed as storage pressure increased; and the growth noted in the AP samples revealed that *S. cerevisiae* could grow at a_W_ 0.971 (even when refrigerated) and sustain it for 28 days. At a_W_ 0.930–0.950, however, curves overlap and smaller load variations were observed; growth was seen in the AP/RT samples during the 14 days but inactivation (though at different rates) was seen after; and, the 50–75 MPa curves, which showed the greatest inactivation results, especially at 50 MPa for a_W_ 0.930 WJ, with at least 3.88 log CFU/mL reduced after 14 days.

At the end of HS, inactivation varied between 0.45 log CFU/mL (a_W_ 0.950 WJ after 10 days at 25 MPa) and at least 4.77 log CFU/mL (a_W_ 0.971 WJ stored at 50 MPa for 21 days).

While growth occurred in samples at all a_W_ levels, it was at a_W_ 0.971 that it reached the greatest values and remained stable throughout storage. This matches expectations since *S. cerevisiae*’s minimum a_W_ growth limit is 0.90 [16], implying that all a_W_ levels tested should allow growth. Additionally, at 25–50 MPa, inactivation rates were higher when a_W_ was lowered, and only at 75 MPa did the results match previous expectations of increased inactivation at higher a_W_ levels, as reported for HPP [17].

To the best of the authors’ knowledge, there is no published research testing the effects of a_W_ variation in *S. cerevisiae* behavior in foods stored by HS. However, the authors of a study that treated water–glycerol mixtures at different levels (a_W_ 0.43–0.99) by HPP (100–600 MPa, 10 min, 25 °C) discovered that complete inactivation (8 log CFU/mL) was obtained at different a_W_/pressure conditions: 0.84/600 MPa, 0.96/400–500 MPa and 0.99/300 MPa [32]. This result demonstrates the effect of increased pressure resistance at lower a_W_ levels, contrasting with the increased inactivation of a_W_ 0.930–0.950 WJ when stored at 25–50 MPa observed in the present work.

In the absence of available HS works targeting *S. cerevisiae*, the findings of studies that have analyzed the variation of yeasts and moulds (YM) (as a group) during storage may provide useful insights. The inactivation of YM in WJ preserved by HS/RT to levels below the DL has been reported [18,33,34,35,36,37], and some authors have described maximum inactivation or a quicker reduction to levels below the DL obtained at the highest storage pressure level. This occurred when WJ (pH 6.23) was preserved for up to 10 days at 50–100 MPa/RT (18–23 °C) and YM counts were reduced to levels below the DL, obtaining a reduction of, at least, 2.55 log CFU/mL after 4 days at 100 MPa or 7 days at 75 MPa, while at 50 MPa, inactivation did not occur [18]. In WJ (pH 6.5) preserved by HS (15 °C, up to 58 days), the authors noticed a reduction to levels below the DL of at least 2.34 log CFU/mL after 14 days at 75 MPa and 58 days at 62.5 MPa, while at 50 MPa, growth was observed at the 7th day (last sampling point) [37]. This effect was described for other products such as raw milk [19], cow and goat fresh cheese [38], ready-to-eat fish soup [39], and pork meat in pieces [40].

The behavior of YM present in refrigerated WJ has also been studied. When citrulline-enriched acidified WJ (pH 3.80) was thermally pasteurized (40–90 s at 80 °C) and stored at 4 °C for 30 days, the initial load of 1.25 and 1.64 log CFU/mL (for the 40 and the 90 s pasteurization, respectively) increased by 4.90 and 4.26 log CFU/mL, respectively, during storage [41]. For WJ (pH 5.34–5.35) processed by UV-C at different intensities and stored at 5 °C for 37 days, YM counts decrease around 1 log CFU/mL (from 2.94 and 2.48 log CFU/mL, for 2.7 and 37.5 J/mL, respectively) until the 13th day (for 2.7 J/mL) or the 17th day (for 37.5 J/mL); however, after that, counts increased around 5 and 3 log CFU/mL for 2.7 and 37.5 J/mL respectively [42]. A third study stored thermally pasteurized (87.7 °C for 20 s) acidified WJ (pH 3.80) at 4 or 8 °C for 30 days. After a decrease in YM counts from 2.18 log CFU/mL to levels below the DL after 10 days, an increase to 1.76 and 1.92 log CFU/mL was noted for WJ stored at 4 and 8 °C, respectively [43]. A different work, testing the same storage and pasteurization conditions as the previous study, analyzed the variation in the yeasts and filamentous fungi group in acidified WJ (pH 3.80) during cold storage of YM and reported a gradual increase in counts of around 4 log CFU/mL at the 30th day, reaching around 6 log CFU/mL [44]. Conversely, when WJ (pH 5.40) was stored for 14 days at 4 °C after being thermally pasteurized for 1–15 min at 80 °C, YM counts dropped to levels below the DL and remained there during storage [45].

Most of these studies described increases in YM counts during refrigeration and, despite not being a direct comparison to the *S. cerevisiae* results (Figure 3), some differences are noticeable, as HS even a_W_ at 0.971 (the typical WJ level) was able to control the yeast’s growth as well as cause its inactivation, albeit at different rates depending on the a_W_/storage pressure condition.

### 3.2. Effect of HS on Inactivation Kinetic Parameters

The impact that the different HS conditions and the different a_W_ levels had on the first-order kinetic model (*D_p_*-values) and Weibull model (*b* and *n*) parameters is presented in Table 4 for *E. coli*, Table 5 for *L. monocytogenes*, and Table 6 for *S. cerevisiae*. The graphical depiction of the model fits and the fitted vs. experimental values plots are provided as Supplementary Material (Figures S2–S10 and Figures S11–S19, respectively).

Table 4 shows that it was possible to obtain a successful fit for curves representing the *E. coli* inactivation by HS using both models, except for the a_W_ 0.950 WJ stored at 75 MPa. The linear model only successfully fitted four curves (a_W_ 0.950 WJ/25 MPa, a_W_ 0.971 WJ/75 MPa, and AP/RF at a_W_ 0.930–0.950) and, in these cases, the Weibull model showed a better fit than the first-order model, revealing higher *Adj-R*^2^ and lower *RMSE* values.

Since there were only a few cases of a good fit of the first-order model to the *E. coli* inactivation data (Table 4), the ability to infer from these results is limited. However, a look into both *D_p_*-values that it was possible to calculate, reveals that lowering the WJ’s a_W_ from 0.971 to 0.950 and storing it at a lower pressure level (25 MPa) allowed a considerable decrease in *D_p_* from 4.24 ± 0.33 days (a_W_ 0.971 WJ/75 MPa) to 1.14 ± 0.31 days (a_W_ 0.950 WJ/25 MPa). This result, along with the lower *D_p_* observed for the AP/RF samples at a_W_ 0.930 (than at a_W_ 0.950), supports the trend observed in Figure 1 that lowering the a_W_ had a stronger inactivating impact than increasing the storage pressure (at the levels evaluated in this work). The *z_p_* was not calculated for the different a_W_ levels due to insufficient data points.

The inactivation kinetic data available for HS is limited, and, for a_W_ variation, in particular, is to date nonexistent, as far as the authors are aware. However, one work evaluated the preservation of raw milk by HS/RT (50–100 MPa), and a *D_p_* of 3.7 days at 50 MPa was calculated, which is lower than the value obtained for the inactivation of a_W_ 0.971 WJ stored at 75 MPa (4.24 ± 0.33 days) but considerably higher than the *D_p_* of a_W_ 0.950 WJ/25 MPa (1.14 ± 0.31 days). The same authors also evaluated the total aerobic mesophiles (TAM) counts, obtaining *D_p_*-values of 25.6 days at 75 MPa [19]. The *D_p_*-values for the inactivation of TAM and Enterobacteriaceae during the HS of raw milk at 75 MPa showed great differences, as 18.81–19.81 and 3.50–3.56 days were calculated for TAM and Enterobacteriaceae, respectively [46]. Another work, studying the preservation of fresh cow and goat cheese, obtained *D_p_*-values for TAM of 17.8 days (cow cheese) and 9.9 days (goat cheese) when the foods were stored at 75 MPa; 14.7 days for cow cheese stored at 50 MPa, 11.2 and 4.2 days for goat cheese at 50 and 75 MPa, respectively, for coliforms; and 11.3 and 9.6 days (cow cheese) and 5.4 and 3.5 days (goat cheese) for Enterobacteriaceae during HS at 50 and 75 MPa, respectively [38]. These results often differ from the results of the present work due to the differences between microbial groups and the foods in which they were tested.

The Weibull model parameters reveal clear patterns as the *b*, a parameter associated with the inactivation rate [47], showed increasing trends as the a_W_ was lowered and as storage pressure was increased, except for the a_W_ 0.971 WJ/75 MPa curve; however, in the latter case, since the value of 1 is included in the confidence interval of the *n* parameter (associated with the shape of the curve), a linear kinetics is suggested [12]. All other *n* values obtained for the *E. coli* inactivation were below 1, revealing an upward concave curve, which indicates a mixed pressure-resistance pressure, with a faster inactivation of the most sensitive subpopulation followed by a slower and steadier decline of the more resistant subpopulation [12,47].

The observed increase in *b* as the storage pressure was increased was also described for apple juice treated by HPP (300–600 MPa, 0–7 min, 21 °C) [48] and contrasts with the decrease in *b* reported in another study where apple juice was treated by HPP (400–475 MPa, 5 °C) [49]. Similarly to Table 4, *n* < 1 values were reported for almond powder stored for 12 months at either −20, 4, or 24 °C and different atmospheres [50] and for pressure levels above 300 MPa for apple juice treated by HPP (300–600 MPa, 0–7 min, 21 °C) [48]. However, another work found only *n* > 1 values when apple juice was processed by HPP (400–475 MPa, 5 °C) [49].

Table 5 presents the data related to the inactivation of *L. monocytogenes* and reveals that successful fits of the models were obtained for most curves. Additionally, the first-order model only successfully fitted the a_W_ 0.950 WJ/25 MPa, a_W_ 0.971 WJ/75 MPa, and the AP/RT at a_W_ 0.930–0.950 curves. A better fit was verified for the Weibull model for a_W_ 0.950 WJ/25 MPa and a_W_ 0.971 WJ/75 MPa, as shown by the higher *Adj-R*^2^ and lower *RMSE* values.

The successful fits of the application of the first-order model to the *L. monocytogenes* HS curves (Table 5) generated similar *D_p_*-values (2.70 ± 0.40 and 2.99 ± 0.54 days for a_W_ 0.971 WJ/75 MPa and a_W_ 0.950 WJ/25 MPa, respectively), suggesting a similar impact between lowering the a_W_ (and storing at a lower pressure level) and increasing the storage pressure without altering the a_W_. Conversely, for the AP/RT controls, lowering the a_W_ to 0.930 or 0.950 resulted in a comparable *D_p_* (1.06 ± 0.19 and 1.23 ± 0.25 days for a_W_ 0.930 and 0.950, respectively).

The behavior of *L. innocua* inoculated in raw milk preserved by HS/RT was analyzed, and *D_p_*-values of 8.6 and 4.5 days were calculated for 50 and 75 MPa, respectively. These values are considerably higher than those in Table 5, for example, the 2.70 ± 0.40 days obtained for a_W_ 0.971 WJ stored at 75 MPa [19].

The *b* values for the *L. monocytogenes* curves increased as storage pressure increased and, as a_W_ was lowered, this trend was also noted (where such comparisons were possible) or, at least, similar values were presented (Table 5). One example is the higher *b* value obtained, at 75 MPa, for a_W_ 0.950 than for a_W_ 0.971 (2.331 ± 0.162 and 0.845 ± 0.101, respectively). The *n* parameter for these curves was always below 1, suggesting an adaptation of the most resistant subpopulation, following the initial inactivation.

Similarly to the present work, the increase in *b* when storage pressure rose was reported in several studies, including apple juice [49], UHT whole milk [51], and whey protein suspensions [52]. The inactivation of *L. monocytogenes* inoculated in tryptic soy agar plates by gaseous chlorine dioxide (150–350 ppm) was evaluated at different a_W_ levels (0.429–0.994), and minor differences in *n* values (below 1) were obtained [53]. Additionally, *n* < 1 values were obtained for the AP/RT and AP/RF storage of avocados [54], enoki, and wood ear mushrooms [55], and Honey Crisp and Fuji apples [56]. For low-salt soft and cured cheeses stored at AP/RT (22 °C/84 days) or AP/RF (4 °C/118 d), however, apart from the refrigerated low-salt soft cheese (*n* < 1), the obtained *n* results were near 1 (suggesting a linear kinetic) [57].

The kinetic parameters for the inactivation of *S. cerevisiae* in WJ at different a_W_ levels are presented in Table 6. A good fit for either the first-order model or the Weibull model was obtained for the 50–75 MPa curves, but not for the 25 MPa curve, due to the growth observed. The Weibull model showed higher *Adj-R*^2^ and lower *RMSE* values, providing better fits. A greater number of successful fits considering both models was possible for *S. cerevisiae* compared to the *E. coli* and *L. monocytogenes* results.

*S. cerevisiae*’s results show the higher inactivating rates of the a_W_ 0.971 WJ/75 MPa and a_W_ 0.930 WJ/50 MPa curves, as they present the lowest *D_p_*-values (2.08 ± 0.20 and 2.23 ± 0.38 days, respectively). At 75 MPa, the *D_p_*-values were also increased as a_W_ was lowered, especially from 0.971 to 0.950, matching previous expectations of increased pressure resistance at lower a_W_ levels.

Despite the absence of *S. cerevisiae* inactivation HS kinetic published data, a work measuring YM inactivation (as a group) with cow and goat fresh cheese preserved by HS/RT (50–100 MPa), obtained *D_p_*-values of 4.7 and 3.9 days for cow and goat, respectively, at 50 MPa [38]. These results are within the variation observed in Table 6 at 50–75 MPa: from 2.08 ± 0.20 days (a_W_ 0.971 WJ/75 MPa) to 7.95 ± 0.94 days (a_W_ 0.930 WJ/75 MPa).

The Weibull model results revealed similar *b* values at a_W_ 0.930, but values increased at 50 MPa for a_W_ 0.950 and at 75 MPa for a_W_ 0.971. The *n* parameter revealed values below, equal to, or above 1, as revealed by the different curve shapes: for a_W_ 0.930–0.950 WJ stored at 75 MPa, the inclusion of 1 suggests that their inactivation curves may be two cases of a linear kinetic. The remaining curves present *n* < 1 for a_W_ 0.950 and *n* > 1 values for a_W_ 0.930 and 0.971, signaling a change in the shape of the curve. An *n* value above 1 indicates that the cumulative damage caused by the exposure to pressure progressively weakened the surviving subpopulation, triggering a faster inactivation [12,58].

Similarly to what was observed for a_W_ 0.971 (Table 6), two works that analyzed *S. cerevisiae* ascospores in beer processed by HPP found an increase in the *b* parameter as the pressure level rose: in beer with different alcohol content (0.0–7.0%) treated at 200–400 MPa for up to 40 min at temperatures not exceeding 36 °C [59]; and, the second, in beer processed by HPP (200–400 MPa, up to 60 min, 23 °C) [60]. These two works also reported only *n* < 1 values [59,60], different from what was described for the inactivation of *S. cerevisiae* by a pulsed electric field treatment on grape juice (40 °C, 34–92 µs), since a change from *n* > 1 to *n* < 1 values was detected when the electric field strength was increased (9–27 kV/cm^2^) [61].

The kinetic parameters derived from the first-order and Weibull models provided quantitative insights on how HS at different a_W_ levels influences microbial inactivation along storage. In general, reductions in a_W_ accelerated microbial inactivation, with this trend suggesting that higher limited water availability amplifies pressure-induced physiological stress, likely by compromising membrane integrity, stability of membrane proteins, and cellular repairing processes [62].

## 4. Conclusions

The research presented in this work demonstrated that HS could control the growth of *E. coli*, *L. monocytogenes*, and *S. cerevisiae*, and that, simultaneously, inactivation often also occurred during storage. HS’s impact on microbial growth control and inactivation was influenced by WJ’s a_W_ level, the microorganism, and the storage pressure. Lowering the a_W_ allowed overall increased inactivation rates, while most growth cases occurred for WJ at a_W_ 0.971, indicating the higher perishability of WJ’s typical a_W_. Also, as expected, higher storage pressures increased inactivation rates. The microbial inactivation kinetic models applied provided overall good fits for the inactivation curves, especially the Weibull model, confirming the microbial inactivation tendencies described above (overall higher *b* values for increased pressure and lower a_W_ levels, for example) and noting differences in microbial resistance: lowest *D_p_*-values were estimated for *E. coli*, followed by *L. monocytogenes* for a_W_ 0.950 WJ stored at 25 MPa and, contrastingly, for *S. cerevisiae,* followed by *L. monocytogenes* and *E. coli* for WJ at a_W_ 0.971 stored at 75 MPa.

The findings presented in this work show that lowering the a_W_ can facilitate microbial growth control and inactivation and support HS’s potential as a food preservation methodology, due to the microbial growth control and inactivation effects observed during storage. These results were obtained at RT and showed that HS could overall preserve WJ better than RF, without requiring temperature control, making HS environmentally friendlier, due to having almost no energy costs and lower greenhouse gas emissions. The impact of the strategies applied in this study was evaluated for microbial parameters; however, their impact on physicochemical, quality, and sensory attributes must also be studied to ensure that characteristic WJ properties are also maintained. Further research is also needed to understand the mechanisms underlying the impact of lowering the a_W_ and how HS’s effects on microorganisms may differ at different a_W_ levels.

## Figures and Tables

**Table 1:** Initial *Escherichia coli* counts, expressed in log CFU/mL, inoculated in the watermelon juice (WJ) at different water activity (a_W_) levels preserved by hyperbaric storage (HS, 25–75 MPa) at room temperature (RT, 18–23 °C) and the atmospheric pressure (AP) controls at RT and refrigerated (RF) conditions presented in Figure 1.

a_W_	Hyperbaric Storage (MPa)			Atmospheric Pressure	
	25	50	75	RT	RF (4 °C)
**0.930**	6.10 ± 0.03	5.98 ± 0.05	6.62 ± 0.08	5.93 ± 0.11	5.93 ± 0.11
**0.950**	5.49 ± 0.08	5.36 ± 0.11	6.48 ± 0.13	6.59 ± 0.02	5.23 ± 0.02
**0.971**	8.02 ± 0.08	7.93 ± 0.05	8.17 ± 0.18	8.21 ± 0.26	8.20 ± 0.03

**Table 2:** Initial *Listeria monocytogenes* counts, expressed in log CFU/mL, inoculated in the watermelon juice (WJ) at different water activity (a_W_) levels preserved by hyperbaric storage (HS, 25–75 MPa) at room temperature (RT, 18–23 °C) and the atmospheric pressure (AP) controls at RT and refrigerated (RF) conditions presented in Figure 2.

a_W_	Hyperbaric Storage (MPa)			Atmospheric Pressure	
	25	50	75	AP/RT	AP/RF (4 °C)
**0.930**	6.51 ± 0.07	6.24 ± 0.07	6.44 ± 0.12	6.51 ± 0.09	6.51 ± 0.09
**0.950**	6.46 ± 0.04	6.11 ± 0.05	6.80 ± 0.26	6.16 ± 0.15	6.16 ± 0.15
**0.971**	7.55 ± 0.04	6.50 ± 0.07	6.27 ± 0.03	7.40 ± 0.08	6.57 ± 0.08

**Table 3:** Initial *Saccharomyces cerevisiae* counts, expressed in log CFU/mL, inoculated in the watermelon juice (WJ) at different water activity (a_W_) levels preserved by hyperbaric storage (HS, 25–75 MPa) at room temperature (RT, 18–23 °C) and the atmospheric pressure (AP) controls at RT and refrigerated (RF) conditions presented in Figure 3.

a_W_	Hyperbaric Storage (MPa)			Atmospheric Pressure	
	25	50	75	AP/RT	AP/RF (4 °C)
**0.930**	6.13 ± 0.02	5.93 ± 0.06	5.59 ± 0.03	6.21 ± 0.04	6.20 ± 0.04
**0.950**	6.06 ± 0.04	6.01 ± 0.22	5.82 ± 0.09	6.29 ± 0.06	6.15 ± 0.12
**0.971**	6.25 ± 0.14	6.47 ± 0.02	6.33 ± 0.05	6.34 ± 0.03	6.34 ± 0.03

**Table 4:** Estimated first-order kinetic (*D_p_*) and Weibull (*b* and *n*) parameters (± 95% confidence interval) of the inactivation of *Escherichia coli* in watermelon juice (WJ) at different water activity (a_W_) levels preserved by hyperbaric storage (HS, 25–75 MPa) at uncontrolled room temperature (RT, 18–23 °C) and the atmospheric pressure controls at RT (AP/RT) and refrigerated conditions (AP/RF, 4 °C). *Adj-R*^2^ values in bold represent the best fit between both models.

*Escherichia coli*		First-Order Model Parameters				
a_W_	Storage Condition	*D_p_* (Days)	*R* ^2^	*Adj-R* ^2^	*RMSE*	
0.930	25 MPa/RT	*				
	50 MPa/RT	*				
	75 MPa/RT	*				
	AP/RT	*				
	AP/RF (4 °C)	1.54 ± 0.08	0.996	0.996	0.097	
0.950	25 MPa/RT	1.14 ± 0.31	0.915	0.902	0.3803	
	50 MPa/RT	*				
	75 MPa/RT	*				
	AP/RT	*				
	AP/RF (4 °C)	2.49 ± 0.24	0.988	0.987	0.1071	
0.971	25 MPa/RT	*				
	50 MPa/RT	*				
	75 MPa/RT	4.24 ± 0.33	0.983	0.982	0.2259	
	AP/RT	*				
	AP/RF (4 °C)	*				
***Escherichia coli***		**Weibull model parameters**				
**a_W_**	**Storage condition**	***b***	***n***	***R* ^2^**	***Adj-R* ^2^**	***RMSE***
0.930	25 MPa/RT	2.003 ± 0.058	0.243 ± 0.033	0.999	**0.999**	0.0427
	50 MPa/RT	2.118 ± 0.165	0.298 ± 0.143	0.94	**0.935**	0.252
	75 MPa/RT	2.326 ± 0.105	0.394 ± 0.064	0.98	**0.978**	0.1586
	AP/RT	2.673 ± 0.023	0.155 ± 0.017	0.999(9)	**0.999(9)**	0.0166
	AP/RF (4 °C)	0.470 ± 0.063	1.191 ± 0.084	0.999	**0.999**	0.0458
0.950	25 MPa/RT	1.623 ± 0.148	0.492 ± 0.096	0.993	0.992	0.1083
	50 MPa/RT	1.879 ± 0.088	0.455 ± 0.086	0.983	0.981	0.1284
	75 MPa/RT	*				
	AP/RT	2.472 ± 0.097	0.421 ± 0.073	0.988	0.987	0.1399
	AP/RF (4 °C)	0.347 ± 0.142	1.085 ± 0.258	0.989	**0.987**	0.104
0.971	25 MPa/RT	0.400 ± 0.146	0.474 ± 0.125	0.932	**0.928**	0.1848
	50 MPa/RT	0.946 ± 0.176	0.536 ± 0.075	0.986	**0.984**	0.1931
	75 MPa/RT	0.292 ± 0.109	0.925 ± 0.137	0.984	**0.983**	0.2169
	AP/RT	*				
	AP/RF (4 °C)	*				

“*”: unable to fit to yield an Adj-R^2^ > 0.900.

**Table 5:** Estimated first-order kinetic (*D_p_*) and Weibull (*b* and *n*) parameters (± 95% confidence interval) of the inactivation of *Listeria monocytogenes* in watermelon juice (WJ) at different water activity (a_W_) levels preserved by hyperbaric storage (HS, 25–75 MPa) at uncontrolled room temperature (RT, 18–23 °C) and the atmospheric pressure controls at RT (AP/RT) and refrigerated conditions (AP/RF, 4 °C). *Adj-R*^2^ values in bold represent the best fit between both models.

*Listeria monocytogenes*		First-Order Model Parameters				
a_W_	Storage	*D_p_* (Days)	*R* ^2^	*Adj-R* ^2^	*RMSE*	
0.930	25 MPa/RT	*				
	50 MPa/RT	*				
	75 MPa/RT	*				
	AP/RT	1.06 ± 0.19	0.962	0.957	0.2663	
	AP/RF (4 °C)	*				
0.950	25 MPa/RT	2.99 ± 0.54	0.916	0.91	0.2783	
	50 MPa/RT	*				
	75 MPa/RT	*				
	AP/RT	1.23 ± 0.25	0.952	0.945	0.2594	
	AP/RF (4 °C)	*				
0.971	25 MPa/RT	*				
	50 MPa/RT	*				
	75 MPa/RT	2.70 ± 0.40	0.929	0.924	0.3176	
	AP/RT	*				
	AP/RF (4 °C)	*				
***Listeria monocytogenes***		**Weibull model parameters**				
**a_W_**	**Storage**	***b***	***n***	***R* ^2^**	***Adj-R* ^2^**	***RMSE***
0.930	25 MPa/RT	1.354 ± 0.207	0.495 ± 0.113	0.98	**0.978**	0.1784
	50 MPa/RT	1.744 ± 0.199	0.535 ± 0.188	0.943	**0.937**	0.247
	75 MPa/RT	*				
	AP/RT	1.463 ± 0.115	0.637 ± 0.080	0.9962	**0.996**	0.0845
	AP/RF (4 °C)	*				
0.950	25 MPa/RT	0.871 ± 0.191	0.542 ± 0.133	0.96	**0.956**	0.1933
	50 MPa/RT	1.947 ± 0.175	0.432 ± 0.149	0.957	**0.952**	0.2241
	75 MPa/RT	2.331 ± 0.162	0.489 ± 0.102	0.961	**0.958**	0.2414
	AP/RT	1.340 ± 0.046	0.586 ± 0.035	0.999	**0.999**	0.0338
	AP/RF (4 °C)	*				
0.971	25 MPa/RT	*				
	50 MPa/RT	*				
	75 MPa/RT	0.845 ± 0.101	0.653 ± 0.066	0.983	**0.981**	0.1571
	AP/RT	*				
	AP/RF (4 °C)	*				

“*”: unable to fit to yield an Adj-R^2^ > 0.900.

**Table 6:** Estimated first-order kinetic (*Dp*) and Weibull (*b* and *n*) parameters (± 95% confidence interval) of the inactivation of *Saccharomyces cerevisiae* in watermelon juice (WJ) at different water activity (a_W_) levels preserved by hyperbaric storage (HS, 25–75 MPa) at uncontrolled room temperature (RT, 18–23 °C) and the atmospheric pressure controls at RT (AP/RT) and refrigerated conditions (AP/RF, 4 °C). *Adj-R*^2^ values in bold represent the best fit between both models.

*Saccharomyces cerevisiae*		First-Order Model Parameters				
a_W_	Storage Condition	*D_p_* (Days)	*R* ^2^	*Adj-R* ^2^	*RMSE*	
0.930	25 MPa/RT	*				
	50 MPa/RT	2.23 ± 0.38	0.966	0.961	0.282	
	75 MPa/RT	7.95 ± 0.94	0.972	0.97	0.1218	
	AP/RT	*				
	AP/RF (4 °C)	*				
0.950	25 MPa/RT	*				
	50 MPa/RT	*				
	75 MPa/RT	7.51 ± 0.50	0.991	0.99	0.0724	
	AP/RT	*				
	AP/RF (4 °C)	*				
0.971	25 MPa/RT	*				
	50 MPa/RT	6.05 ± 1.15	0.932	0.926	0.2556	
	75 MPa/RT	2.08 ± 0.20	0.989	0.987	0.1654	
	AP/RT	*				
	AP/RF (4 °C)	*				
***Saccharomyces cerevisiae***		**Weibull model parameters**				
**a_W_**	**Storage condition**	***b***	***n***	***R* ^2^**	***Adj-R* ^2^**	***RMSE***
0.930	25 MPa/RT	*				
	50 MPa/RT	0.118 ± 0.084	1.666 ± 0.369	0.993	**0.992**	0.1255
	75 MPa/RT	0.089 ± 0.052	1.123 ± 0.234	0.974	**0.971**	0.1193
	AP/RT	*				
	AP/RF (4 °C)	*				
0.950	25 MPa/RT	*				
	50 MPa/RT	0.459 ± 0.228	0.609 ± 0.213	0.926	**0.92**	0.2526
	75 MPa/RT	0.158 ± 0.040	0.936 ± 0.103	0.992	**0.991**	0.0683
	AP/RT	*				
	AP/RF (4 °C)	*				
0.971	25 MPa/RT	*				
	50 MPa/RT	0.016 ± 0.010	1.874 ± 0.234	0.993	**0.992**	0.0818
	75 MPa/RT	0.295 ± 0.068	1.246 ± 0.122	0.997	**0.997**	0.083
	AP/RT	*				
	AP/RF (4 °C)	*				

“*”: unable to fit to yield an *Adj-R*^2^ > 0.900.

## Data Availability

Data will be made available upon request to the corresponding author.

## Supplementary Materials

The following supporting information can be downloaded at: https://www.mdpi.com/article/10.3390/foods15040741/s1, Table S1: Water activity (a_W_) of watermelon juice (WJ) for different amounts of added sucrose. Figure S1: Linear regression of the measured water activity (a_W_) values after adding sucrose to the watermelon juice (WJ). Figures S2–S10: Graphical representation of the first order and Weibull models; Figures S11–S19: Graphical representation of the fitted versus experimental values.

## Abbreviations

The following abbreviations are used in this manuscript:

*Adj-R*^2^	Adjusted coefficient of determination
ANOVA	Analysis of Variance
AP	Atmospheric pressure
a_W_	Water activity
CFU	Colony-forming unit
DL	Detection limit
*D_p_*	Decimal reduction time
FDA	U.S. Food and Drug Administration
HPP	High-pressure processing
HS	Hyperbaric storage
*N*	Microbial load
*N*_0_	Initial microbial load
QL	Quantification limit
*R*^2^	Coefficient of determination
RF	Refrigeration
*RMSE*	Root mean square error
RT	Room temperature
TAM	Total aerobic mesophiles
UK	United Kingdom
UV-C	Ultraviolet-C
USA	United States of America
WJ	Watermelon juice
YM	Yeast and moulds
YMA	Yeast malt agar
