# Supplementary material for: Water Activity Effect on Microbial Behavior During Hyperbaric Storage at Room Temperature of Watermelon Juice as a Case Study
Source: Foods. 2026 Feb 17;15(4):741. doi: 10.3390/foods15040741 (PMC12939255; doi:10.3390/foods15040741)
Supplement: Supplementary file 1 [file foods-15-00741-s001.zip › foods-4121507-supplementary.pdf]

Supplementary Materials

Table S1 - Water activity (a_W) of watermelon juice (WJ) for different amounts of added sucrose.

Added sucrose (g/100 mL WJ)	a_W
**85**	0.956 ± 0.004
**90**	0.934 ± 0.005
**95**	0.921 ± 0.008
**100**	0.898 ± 0.006

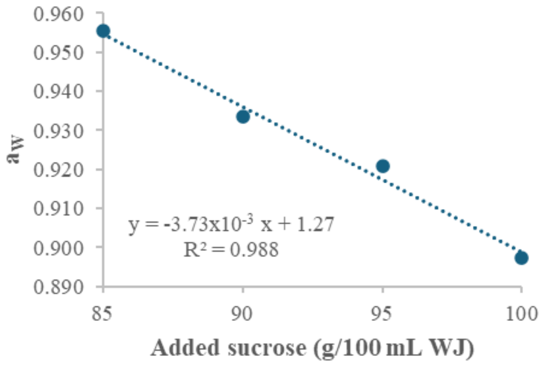

Figure S1 - Linear regression of the measured water activity (a_W) values after adding sucrose to the watermelon juice (WJ).

Graphical representations of the First order kinetics model (*Escherichia coli*):

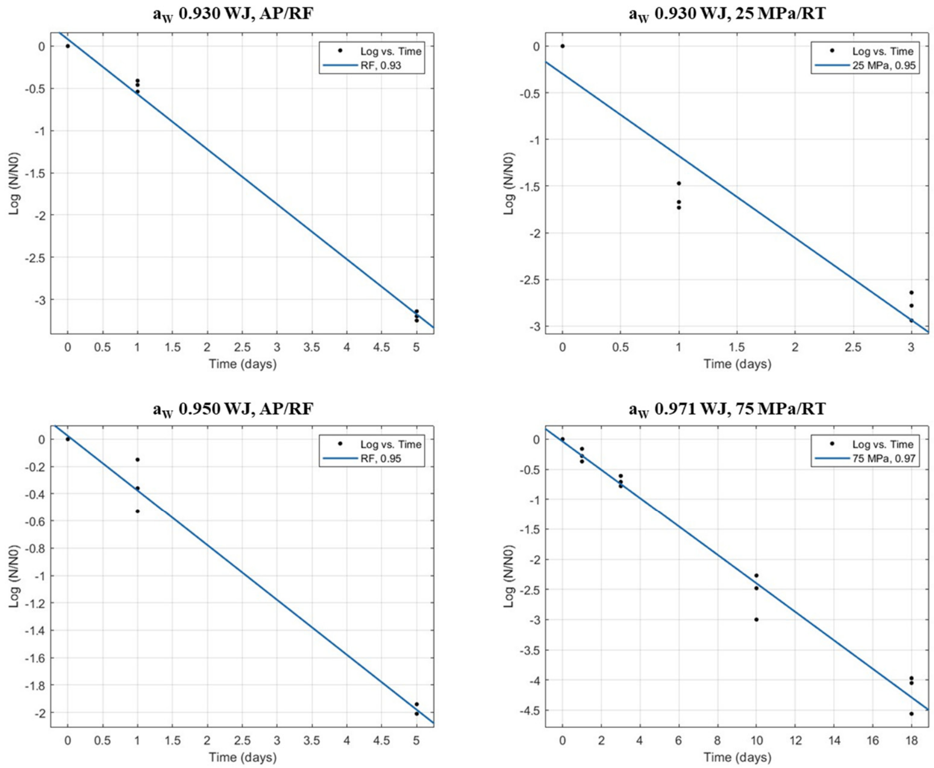

Figure S2 - First order model fitting for *Escherichia coli* inactivation in watermelon juice (WJ) at water activity (a_W) 0.930, 0.950 and 0.971 stored by hyperbaric storage (HS, 25 and 75 MPa) at uncontrolled room temperature (RT, 18-23 °C) and the atmospheric pressure control at refrigerated conditions (AP/RF, 4 °C) (only quantifiable experimental values (above 2.70 log CFU/mL) were used for the model fitting) – data points represent the experimental values, whereas the curve was obtained using the kinetic parameters obtained by the model fitting.

Graphical representations of the Weibull model (*Escherichia coli*):

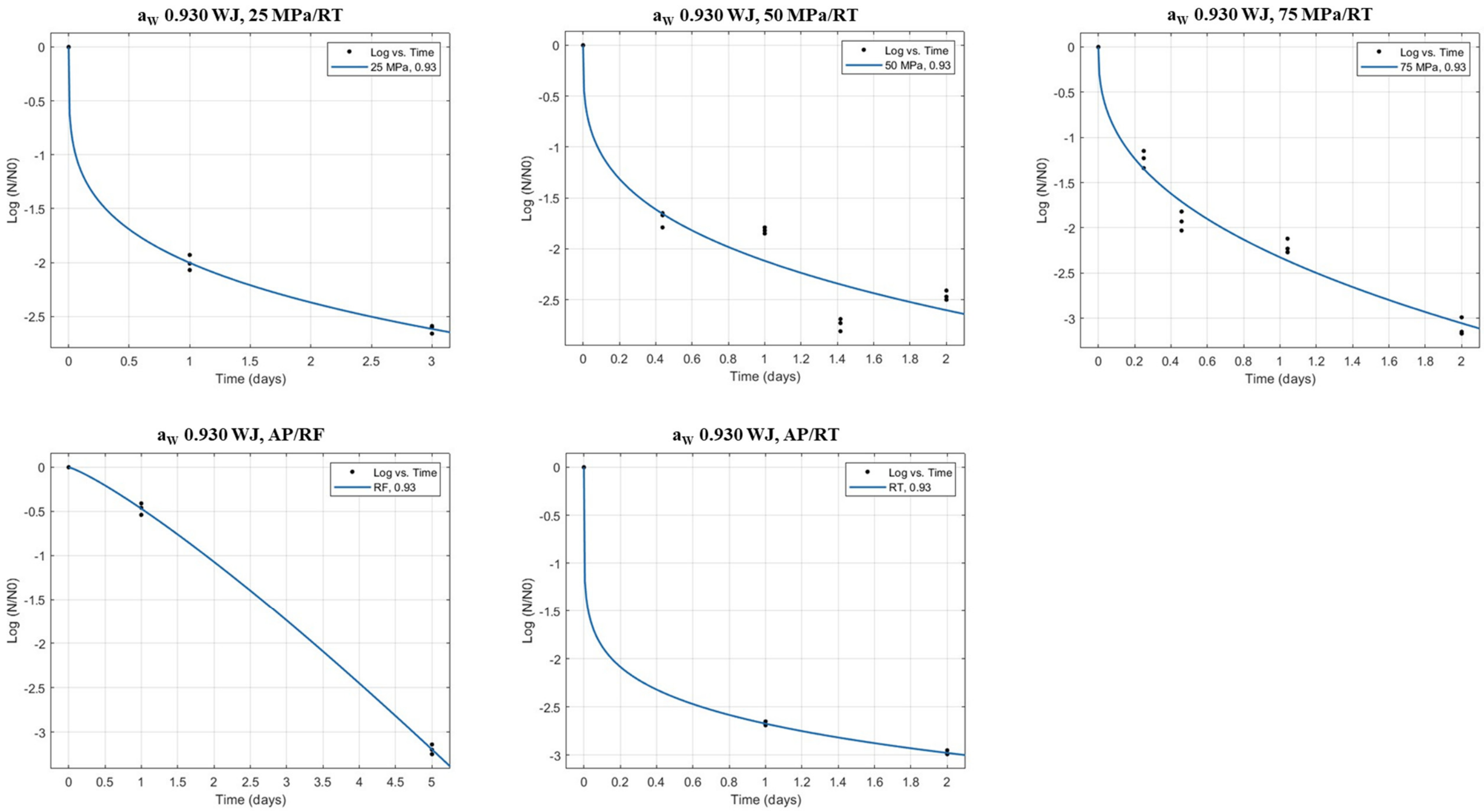

Figure S3 - Weibull model fitting for *Escherichia coli* inactivation in watermelon juice (WJ) at water activity (a_W) 0.930 stored by hyperbaric storage (HS, 25-75 MPa) at uncontrolled room temperature (RT, 18-23 °C) and the atmospheric pressure (AP) control at RT and refrigerated conditions (AP/RF, 4 °C) (only quantifiable experimental values (above 2.70 log CFU/mL) were used for the model fitting) – data points represent the experimental values, whereas the curve was obtained using the kinetic parameters obtained by the model fitting.

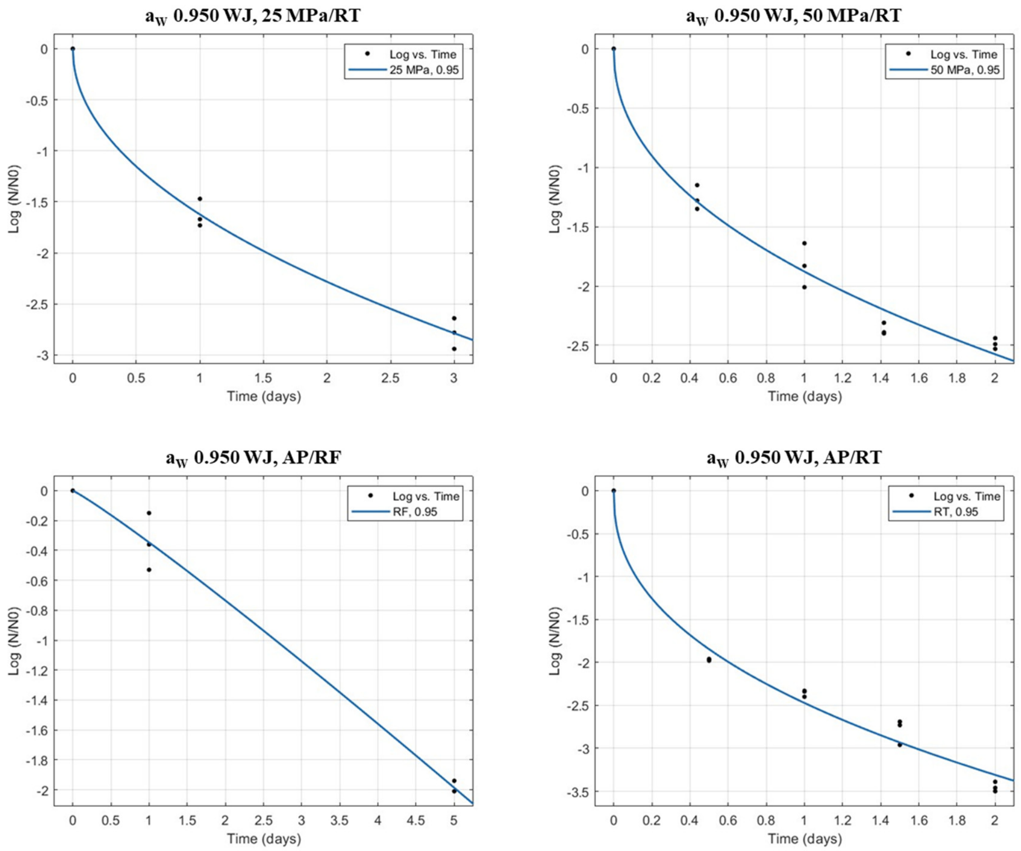

Figure S4 - Weibull model fitting for *Escherichia coli* inactivation in watermelon juice (WJ) at water activity (a_w) 0.950 stored by hyperbaric storage (HS, 25 and 50 MPa) at uncontrolled room temperature (RT, 18-23 °C) and the atmospheric pressure (AP) controls at RT and refrigerated conditions (AP/RF, 4 °C) (only quantifiable experimental values (above 2.70 log CFU/mL) were used for the model fitting) – data points represent the experimental values, whereas the curve was obtained using the kinetic parameters obtained by the model fitting.

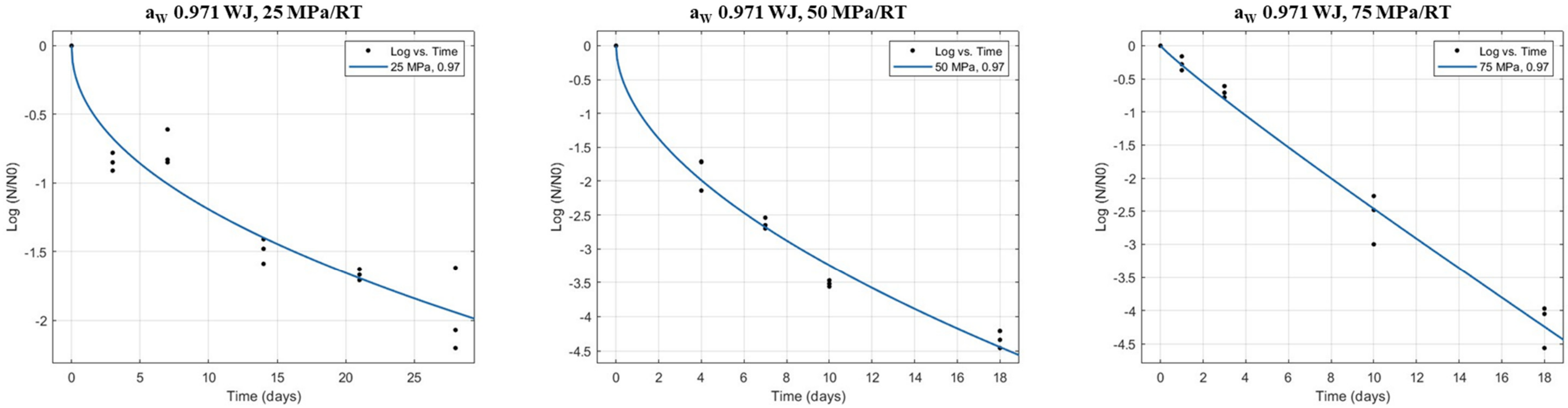

Figure S5 - Weibull model fitting for *Escherichia coli* inactivation in watermelon juice (WJ) at water activity (a_W) 0.971 stored by hyperbaric storage (HS, 25-75 MPa) at uncontrolled room temperature (RT, 18-23 °C) (only quantifiable experimental values (above 2.70 log CFU/mL) were used for the model fitting) – data points represent the experimental values, whereas the curve was obtained using the kinetic parameters obtained by the model fitting.

Graphical representations of the First order kinetics model (*Listeria monocytogenes*):

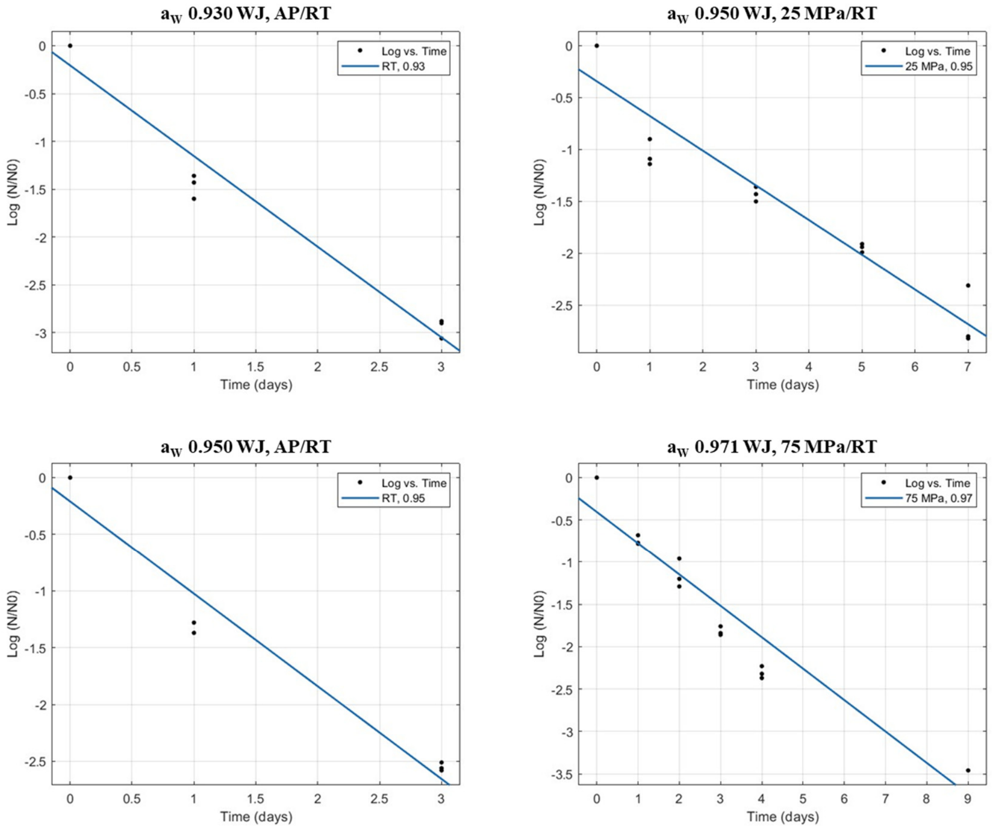

Figure S6 - First order model fitting for *Listeria monocytogenes* inactivation in watermelon juice (WJ) at water activity (a_W) 0.930, 0.950 and 0.971 stored by hyperbaric storage (HS, 25 and 75 MPa) at uncontrolled room temperature (RT, 18-23 °C) and the atmospheric pressure (AP) controls at RT (only quantifiable experimental values (above 2.70 log CFU/mL) were used for the model fitting) – data points represent the experimental values, whereas the curve was obtained using the kinetic parameters obtained by the model fitting.

Graphical representations of the Weibull model (*Listeria monocytogenes*):

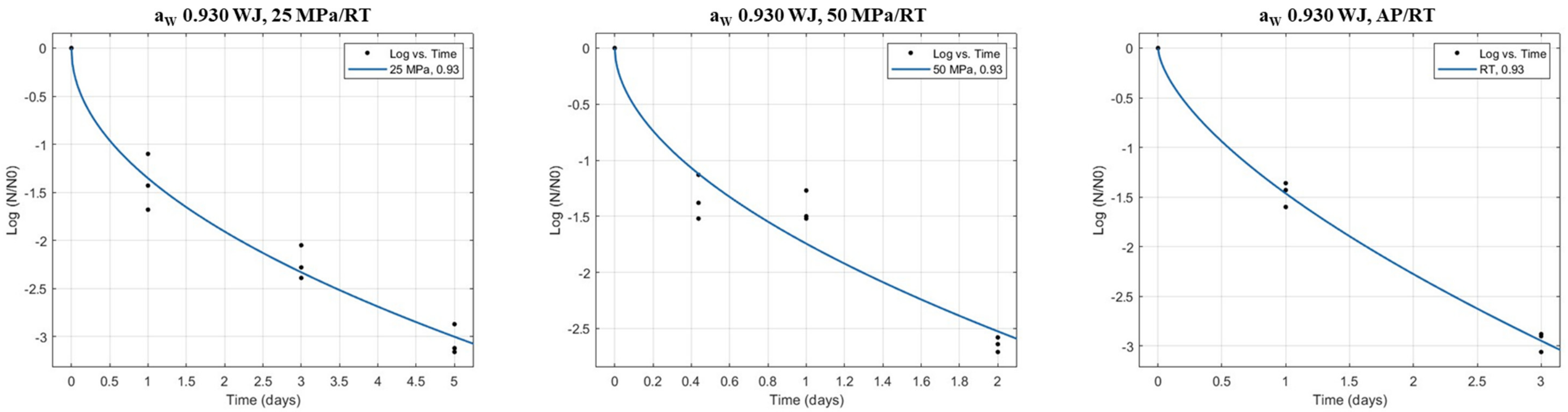

Figure S7 - Weibull model fitting for *Listeria monocytogenes* inactivation in watermelon juice (WJ) at water activity (a_W) 0.930 stored by hyperbaric storage (HS, 25 and 50 MPa) at uncontrolled room temperature (RT, 18-23 °C) and the atmospheric pressure (AP) control at RT (only quantifiable experimental values (above 2.70 log CFU/mL) were used for the model fitting) – data points represent the experimental values, whereas the curve was obtained using the kinetic parameters obtained by the model fitting.

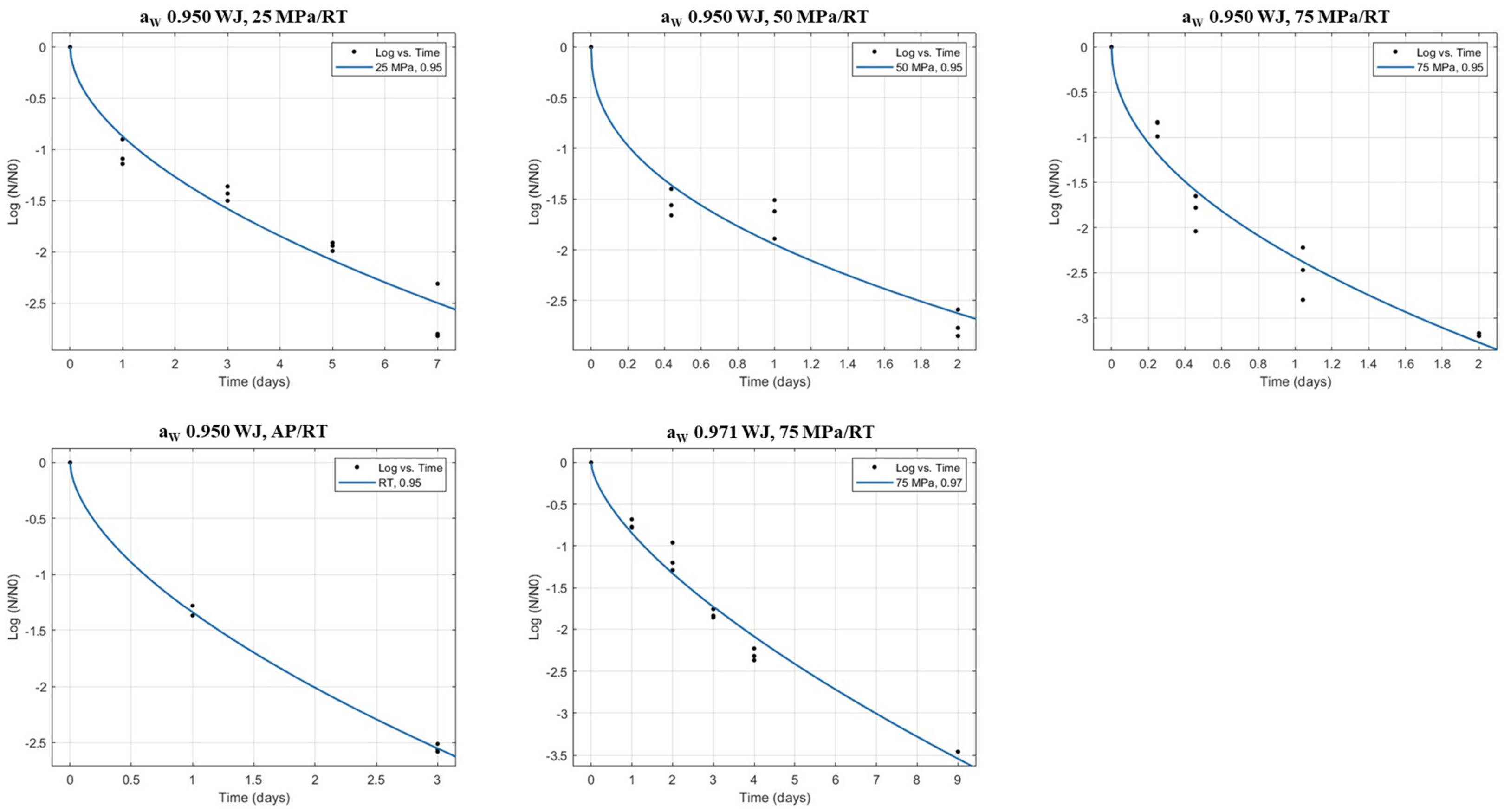

Figure S8 - Weibull model fitting for *Listeria monocytogenes* inactivation in watermelon juice (WJ) at water activity (a_w) 0.950 and 0.971 stored by hyperbaric storage (HS, 25-75 MPa) at uncontrolled room temperature (RT, 18-23 °C) and the atmospheric pressure (AP) control at RT (only quantifiable experimental values (above 2.70 log CFU/mL) were used for the model fitting) – data points represent the experimental values, whereas the curve was obtained using the kinetic parameters obtained by the model fitting.

Graphical representations of the First order kinetics model (*Saccharomyces cerevisiae*):

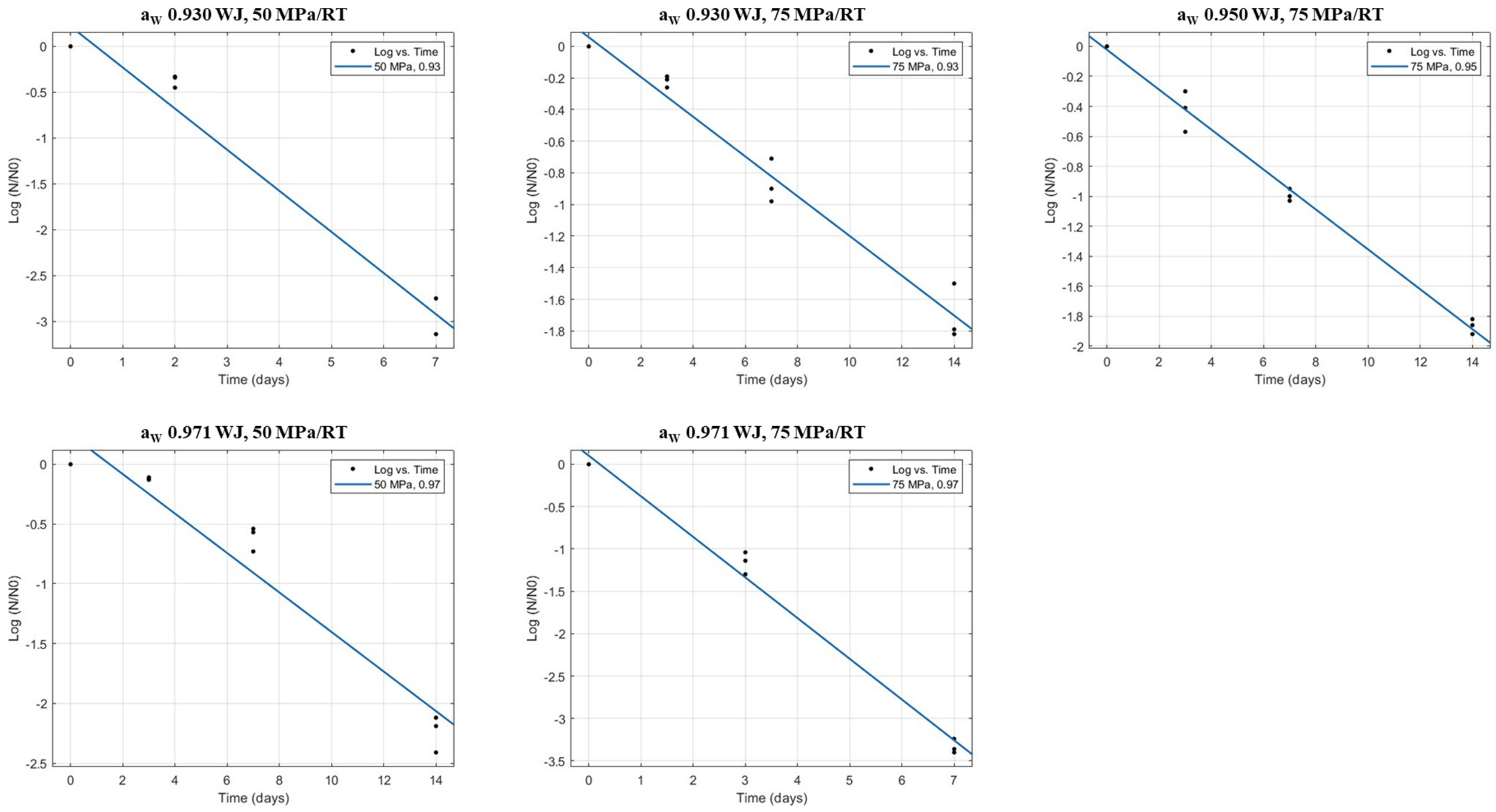

Figure S9 - First order model fitting for *Saccharomyces cerevisiae* inactivation in watermelon juice (WJ) at water activity (a_w) 0.930, 0.950 and 0.971 stored by hyperbaric storage (HS, 50 and 75 MPa) at uncontrolled room temperature (RT, 18-23 °C) (only quantifiable experimental values (above 2.70 log CFU/mL) were used for the model fitting) – data points represent the experimental values, whereas the curve was obtained using the kinetic parameters obtained by the model fitting.

Graphical representations of the Weibull model (*Saccharomyces cerevisiae*):

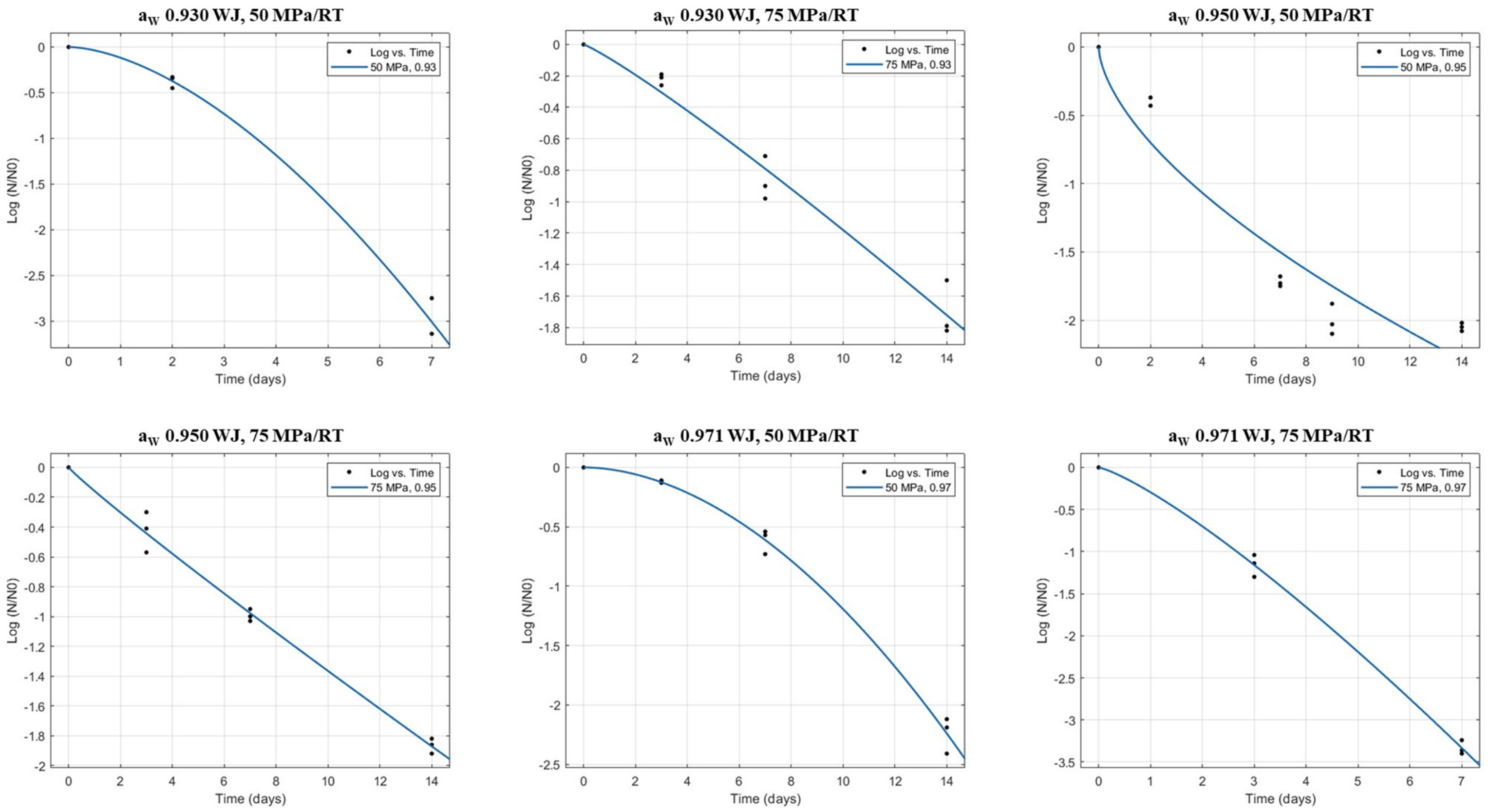

Figure S10 - Weibull model fitting for *Saccharomyces cerevisiae* inactivation in watermelon juice (WJ) at water activity (a_W) 0.930, 0.950 and 0.971 stored by hyperbaric storage (HS, 50 and 75 MPa) at uncontrolled room temperature (RT, 18-23 °C) (only quantifiable experimental values (above 2.70 log CFU/mL) were used for the model fitting) – data points represent the experimental values, whereas the curve was obtained using the kinetic parameters obtained by the model fitting.

Fitted vs experimental value plots for the First order kinetics model (*Escherichia coli*):

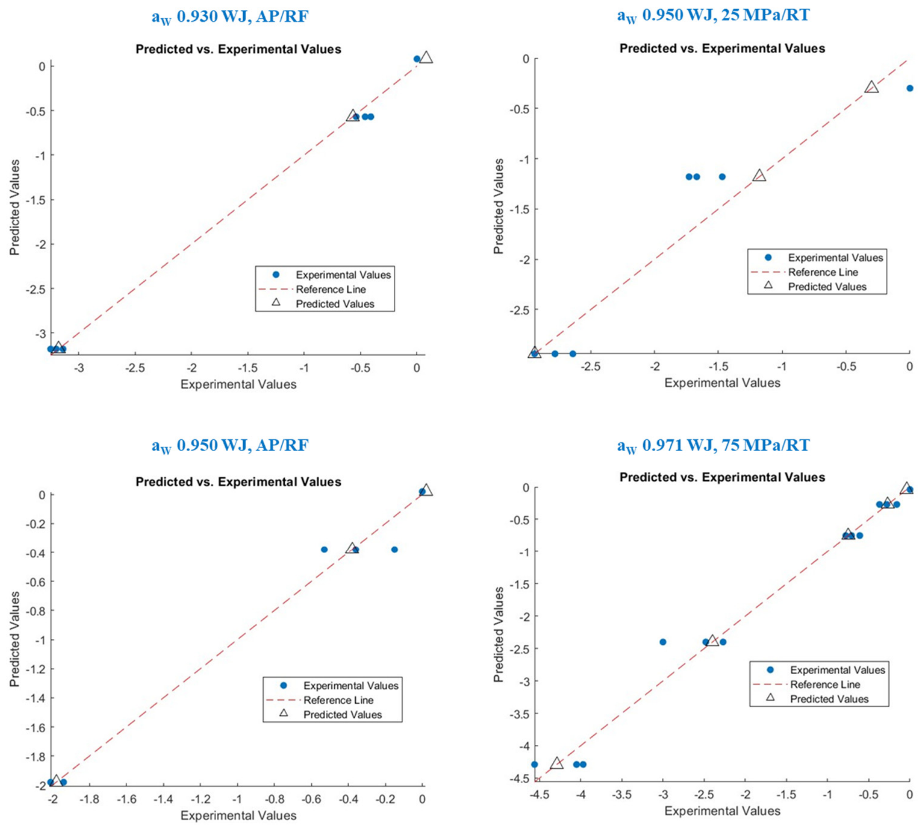

Figure S11 - Experimental versus fitted values derived from the First order kinetics model describing the inactivation of *Escherichia coli* by hyperbaric storage (HS, 25 and 75 MPa) at uncontrolled room temperature (RT, 18-23 °C) and the atmospheric pressure controls at refrigerated conditions (AP/RF, 4 °C) conditions in watermelon juice at water activity (a_w) 0.930, 0.950 and 0.971 (only quantifiable experimental values (above 2.70 log CFU/mL)) were used for the model fitting. The blue circles represent the experimental values, whereas the triangles indicate the values fitted by the model.

Fitted vs experimental value plots for the Weibull model (*Escherichia coli*):

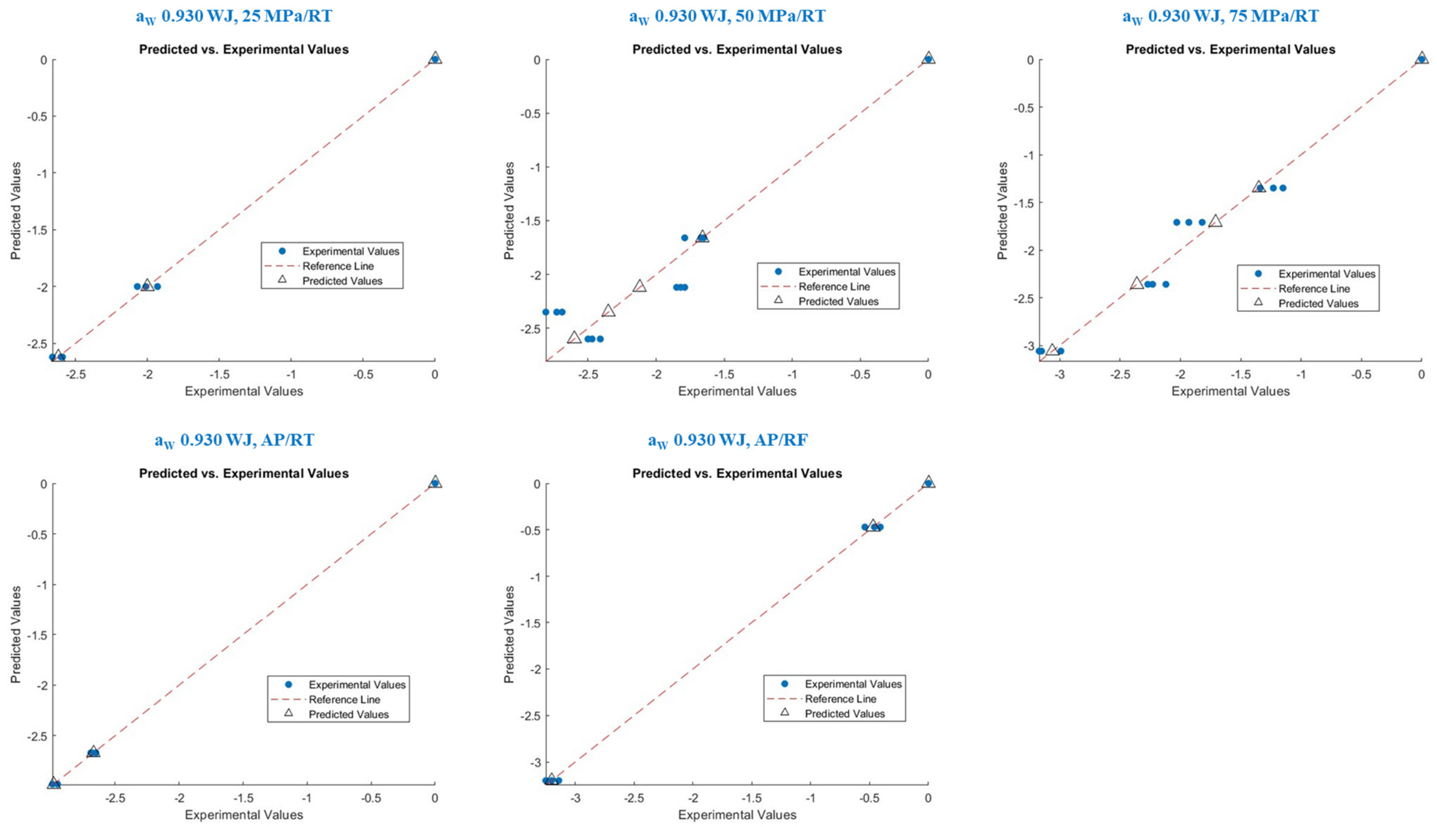

Figure S12 - Experimental versus fitted values derived from the Weibull model describing the inactivation of *Escherichia coli* by hyperbaric storage (HS, 25-75 MPa) at uncontrolled room temperature (RT, 18-23 °C) and the atmospheric pressure (AP) controls at RT and refrigerated conditions (AP/RF, 4 °C) conditions in watermelon juice at water activity (a_W) 0.930 (only quantifiable experimental values (above 2.70 log CFU/mL)) were used for the model fitting. The blue circles represent the experimental values, whereas the triangles indicate the values fitted by the model.

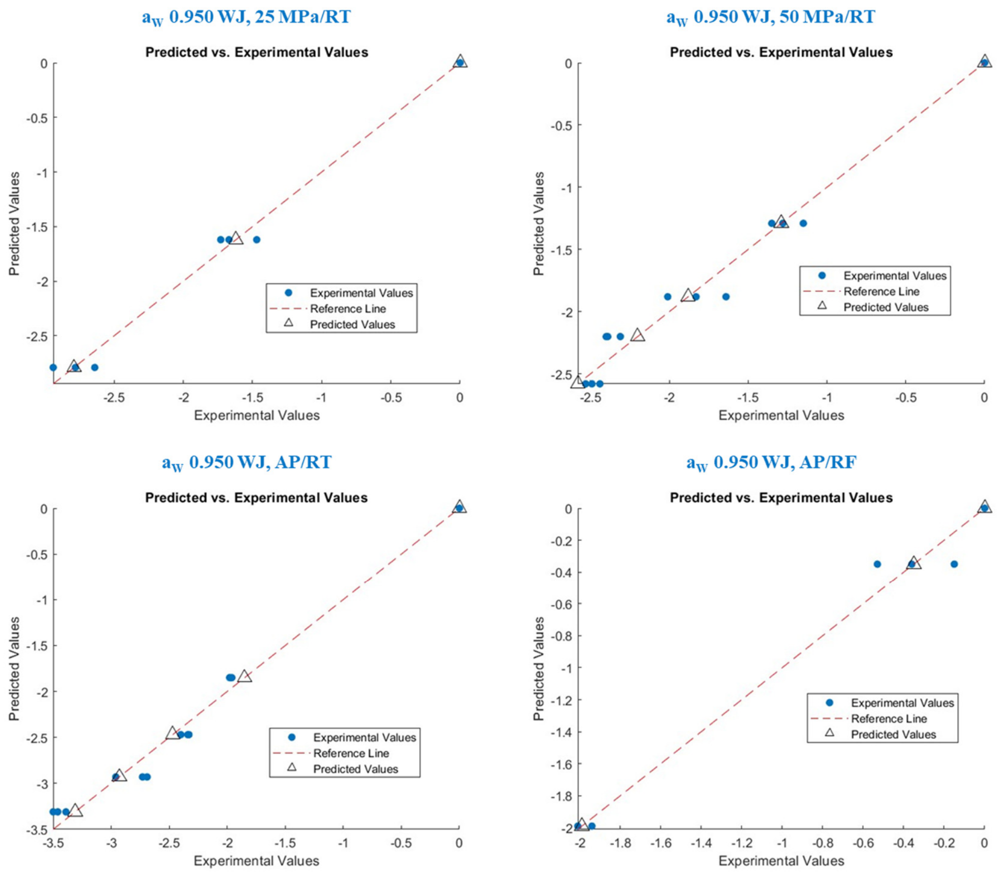

Figure S13 - Experimental versus fitted values derived from the Weibull model describing the inactivation of *Escherichia coli* by hyperbaric storage (HS, 25 and 50 MPa) at uncontrolled room temperature (RT, 18-23 °C) and the atmospheric pressure (AP) controls at RT and refrigerated conditions (AP/RF, 4 °C) conditions in watermelon juice at water activity (a_w) 0.950 (only quantifiable experimental values (above 2.70 log CFU/mL)) were used for the model fitting. The blue circles represent the experimental values, whereas the triangles indicate the values fitted by the model.

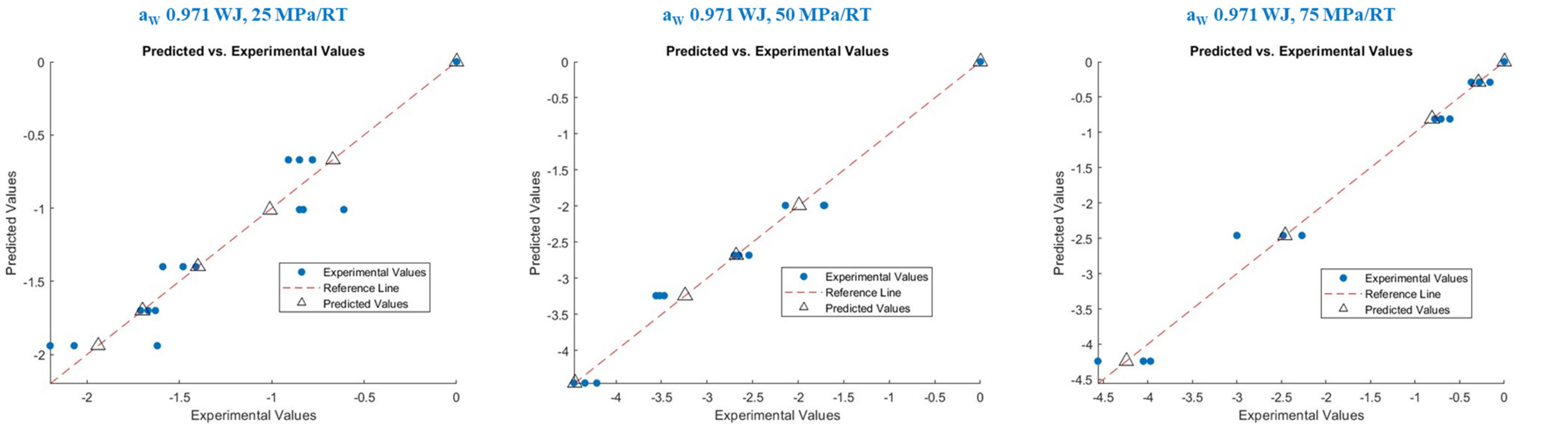

Figure S14 - Experimental versus fitted values derived from the Weibull model describing the inactivation of *Escherichia coli* by hyperbaric storage (HS, 25-50 MPa) at uncontrolled room temperature (RT, 18-23 °C) conditions in watermelon juice at water activity (a_w) 0.971 (only quantifiable experimental values (above 2.70 log CFU/mL)) were used for the model fitting. The blue circles represent the experimental values, whereas the triangles indicate the values fitted by the model.

Fitted vs experimental value plots for the First order kinetics model (*Listeria monocytogenes*):

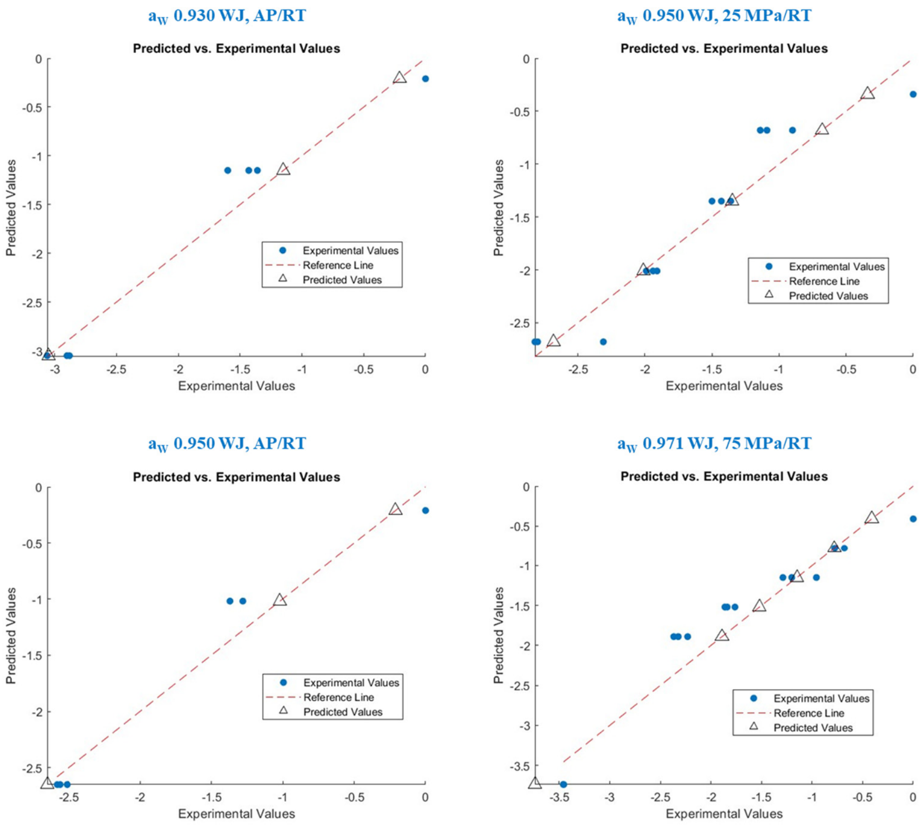

Figure S15 - Experimental versus fitted values derived from the First order kinetics model describing the inactivation of *Listeria monocytogenes* by hyperbaric storage (HS, 25 and 75 MPa) at uncontrolled room temperature (RT, 18-23 °C) and the atmospheric pressure (AP) controls at RT in watermelon juice at water activity (a_W) 0.930, 0.950 and 0.971 (only quantifiable experimental values (above 2.70 log CFU/mL)) were used for the model fitting. The blue circles represent the experimental values, whereas the triangles indicate the values fitted by the model.

Fitted vs experimental value plots for the Weibull model (*Listeria monocytogenes*):

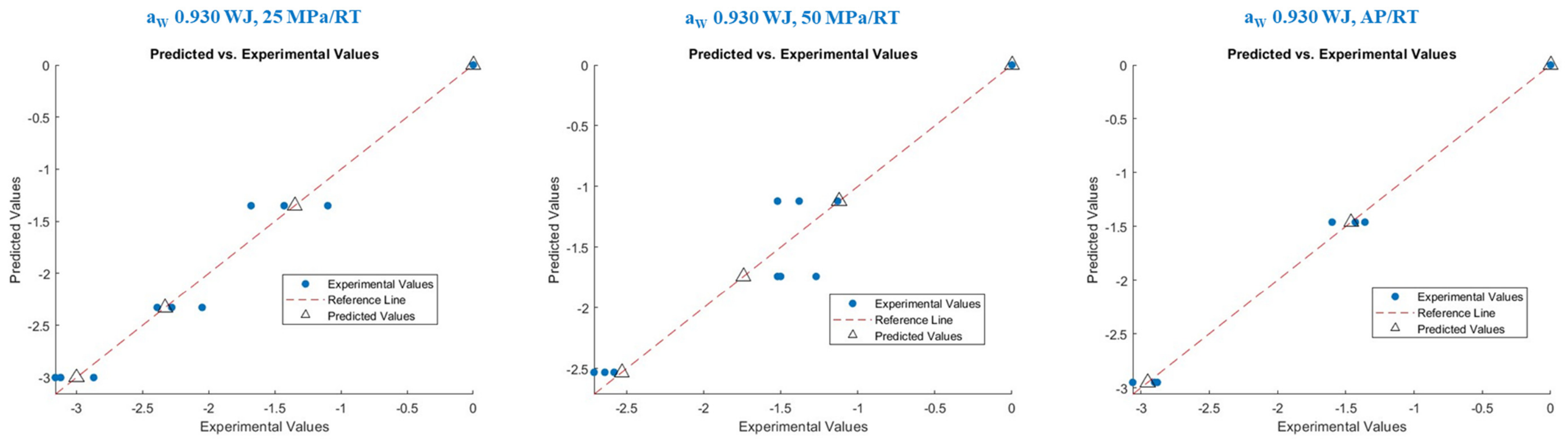

Figure S16 - Experimental versus fitted values derived from the Weibull model describing the inactivation of *Listeria monocytogenes* by hyperbaric storage (HS, 25 and 50 MPa) at uncontrolled room temperature (RT, 18-23 °C) and the atmospheric pressure (AP) control at RT in watermelon juice at water activity (a_w) 0.930 (only quantifiable experimental values (above 2.70 log CFU/mL)) were used for the model fitting. The blue circles represent the experimental values, whereas the triangles indicate the values fitted by the model.

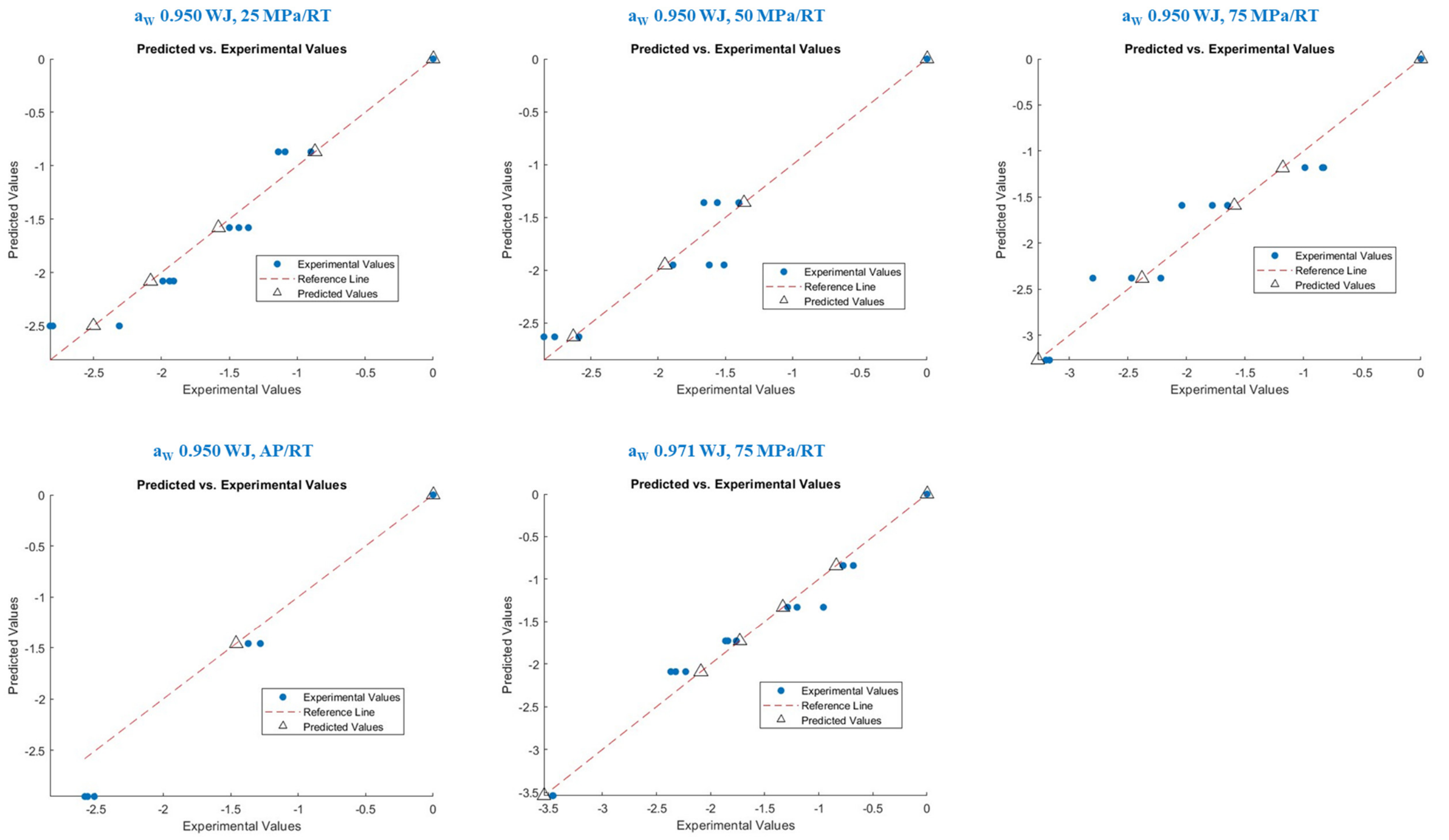

Figure S17 - Experimental versus fitted values derived from the Weibull model describing the inactivation of *Listeria monocytogenes* by hyperbaric storage (HS, 25-75 MPa) at uncontrolled room temperature (RT, 18-23 °C) and the atmospheric pressure (AP) controls at RT in watermelon juice at water activity (a_w) 0.950 and 0.971 (only quantifiable experimental values (above 2.70 log CFU/mL)) were used for the model fitting. The blue circles represent the experimental values, whereas the triangles indicate the values fitted by the model.

Fitted vs experimental value plots for the First order kinetics model (*Saccharomyces cerevisiae*):

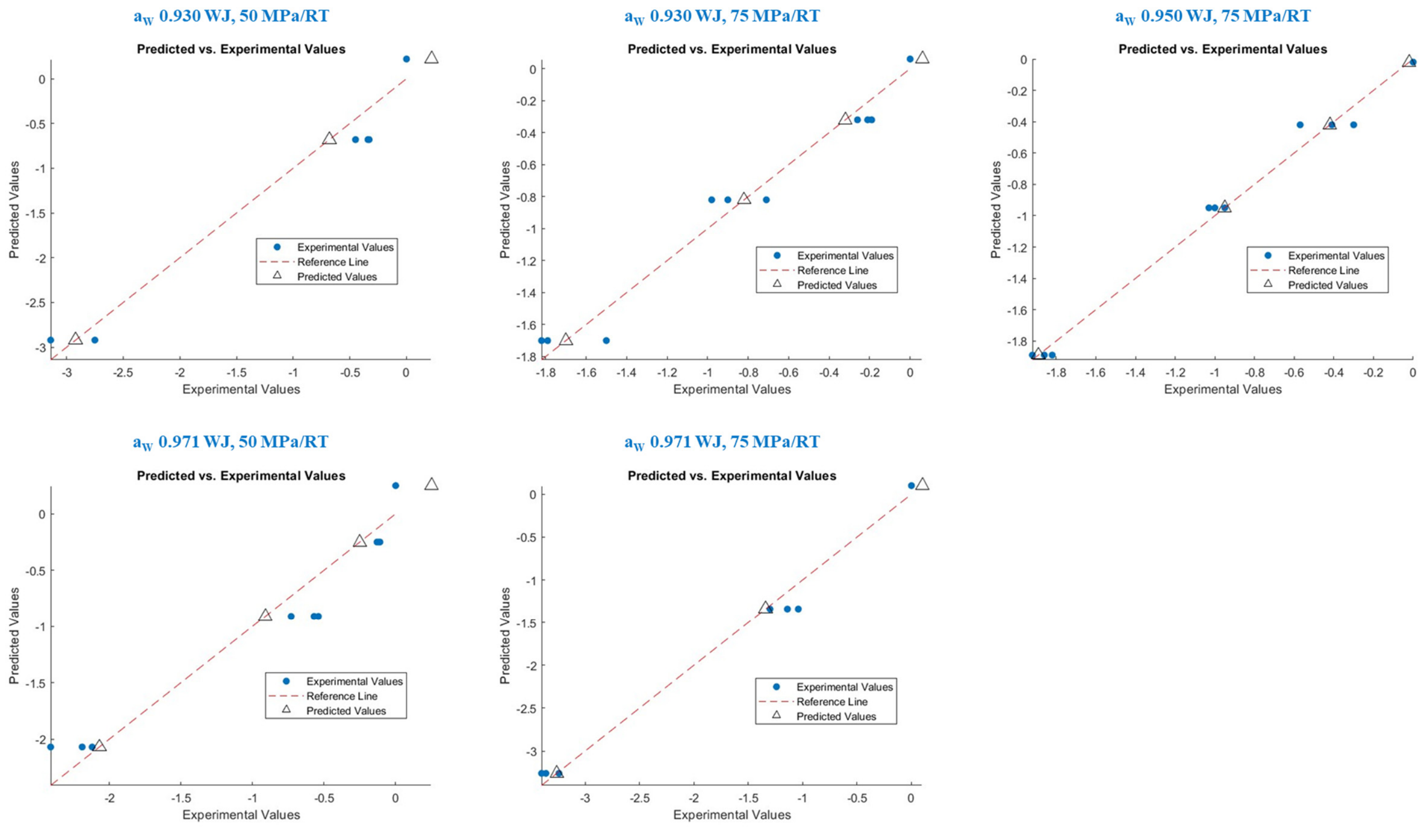

Figure S18 - Experimental versus fitted values derived from the First order kinetics model describing the inactivation of *Saccharomyces cerevisiae* by hyperbaric storage (HS, 50 and 75 MPa) at uncontrolled room temperature (RT, 18-23 °C) in watermelon juice at water activity (a_w) 0.930, 0.950 and 0.971 (only quantifiable experimental values (above 2.70 log CFU/mL)) were used for the model fitting. The blue circles represent the experimental values, whereas the triangles indicate the values fitted by the model.

Fitted vs experimental value plots for the Weibull model (*Saccharomyces cerevisiae*):

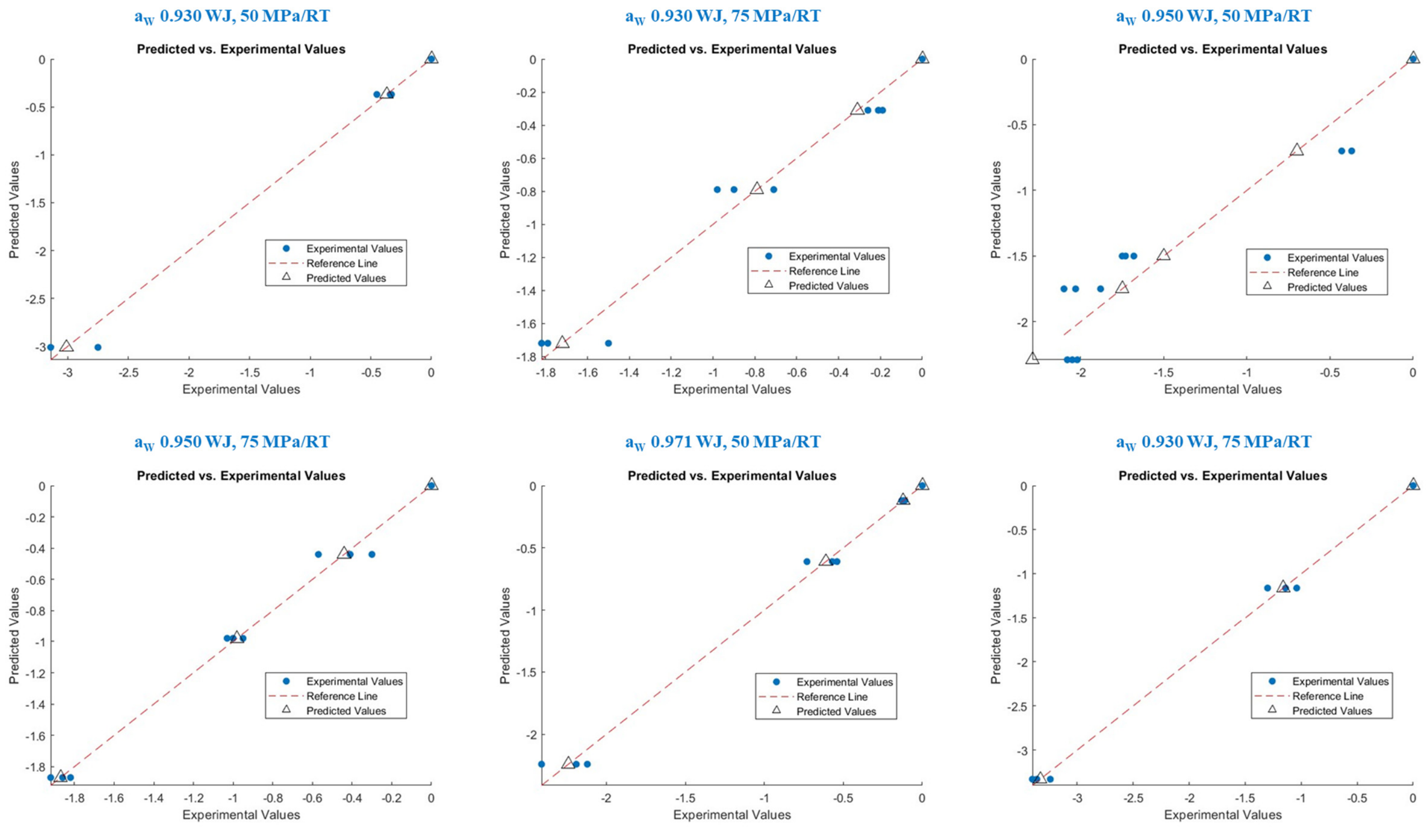

Figure S19 - Experimental versus fitted values derived from the Weibull model describing the inactivation of *Saccharomyces cerevisiae* by hyperbaric storage (HS, 50 and 75 MPa) at uncontrolled room temperature (RT, 18-23 °C) in watermelon juice at water activity (a_w) 0.930, 0.950 and 0.971 (only quantifiable experimental values (above 2.70 log CFU/mL)) were used for the model fitting. The blue circles represent the experimental values, whereas the triangles indicate the values fitted by the model.
